# Prediction of porosity, hardness and surface roughness in additive manufactured AlSi10Mg samples

**DOI:** 10.1371/journal.pone.0316600

**Published:** 2025-03-10

**Authors:** Fatma Alamri, Imad Barsoum, Shrinivas Bojanampati, Maher Maalouf

**Affiliations:** 1 Management Science and Engineering, Khalifa University, Abu Dhabi, UAE; 2 Advanced Digital & Additive Manufacturing Research Centre, Khalifa University, Abu Dhabi, UAE; 3 Mechanical and Nuclear Engineering, Khalifa University, Abu Dhabi, UAE; 4 Department of Engineering Mechanics, Royal Institute of Technology (KTH), Stockholm, Sweden; 5 Digital Supply Chain and Operations Research Center, Khalifa University, Abu Dhabi, UAE; University of Vigo, SPAIN

## Abstract

Despite the advantages of additive manufacturing, its widespread adoption is still hindered by the poor quality of the fabricated parts. Advanced machine learning techniques to predict part quality can improve repeatability and open additive manufacturing to various industries. This study aims to accurately predict the relative density, surface roughness and hardness of AlSi10Mg samples produced by selective laser melting regarding process parameters such as scan speed, layer thickness, laser power, and hatch distance. For this purpose, data including porosity, surface hardness, and roughness were extracted from the literature, and additional measurements were performed on additive manufactured samples in the current work. This work compares five supervised machine learning algorithms, including artificial neural networks, support vector regression, kernel ridge regression, random forest, and Lasso regression. These models are evaluated based on the coefficient of determination and the mean squared error. On the basis of the computational results, the artificial neural network outperformed in predicting relative density, surface roughness, and hardness. Feature importance analysis on the compiled dataset using ANN revealed that laser power and scan speed are the most important features affecting relative density (e.g., porosity) and hardness, while scan speed and layer thickness significantly impact the surface roughness of the parts. The study identified an optimal laser power and scan speed region that achieves a relative density >  99%, surface roughness <  10 µm, and hardness >  120 HV. The results presented in this study provide significant advantages for additive manufacturing, potentially reducing experimentation costs by identifying the process parameters that optimize the quality of the fabricated parts.

## 1. Introduction

Additive Manufacturing (AM) is a process of joining materials to produce objects from 3D model data, commonly layer by layer, in contrast to subtractive manufacturing and machining methods [1–4]. Over the last few decades, public interest in innovation and the improvement of AM technologies has risen dramatically. Compared to traditional subtractive manufacturing methods, AM has been proven to be capable of improving manufacturing complexity, reducing production time and cost, and improving customization levels [5]. However, despite these numerous benefits, the comprehensive implementation of AM techniques has been somewhat overlooked due to concerns surrounding the ultimate quality of the parts, such as long build times, rough surface finish, and inadequate dimensional accuracy [6]. To produce a more attractive product, the process parameters can be adjusted to improve the quality of the fabricated parts.

AM has several processes that are used in applications across a wide range of industries. The AM processes can be divided into three categories based on the raw materials used: AM based on liquids, solids and powders [7]. Laser-based powder metal AM technology has been growing steadily in recent years [8], due to its accuracy rate, dimensional stability, and mechanical strength [9]. This technology has been finding applications in various industries such as manufacturing complex geometries and structures for automotive and aerospace parts, and medical implants [8].

Selective laser melting (SLM) is a type of laser powder bed fusion (L-PBF) that produces metallic parts from powder. In this process, laser beams with high power are used to fuse fine metallic particles layer by layer to create 3D parts. Various parameters such as material qualities, powder properties, and machine specifications affect the characteristics of the fabricated parts. In addition, aspects of the manufacturing process, such as scan speed, laser power, layer thickness, and hatch distance, can have a significant influence on the performance characteristics of parts manufactured using SLM [10]. In addition, the energy density, which is a combination of these process parameters, plays a crucial role in the overall performance of parts manufactured using SLM. The energy density refers to the amount of energy supplied per unit of volume, which can be determined using the formula ED = P/ (v ×  h ×  l). This equation takes into account laser power (P), scan speed (v), hatch distance (h) and layer thickness (l) to quantify the energy density in joules per cubic millimeter (J mm • 3).

A poor combination of parameters might result in inadequate powder fusion, the occurrence of the balling phenomenon, and the formation of keyholes [10]. These mechanisms can influence the mechanical properties and final microstructure of the fabricated parts (e.g., relative density, surface roughness, and hardness). The lack of fusion arises when the substrate is not sufficiently melted to a substantial depth, which causes voids to form in between laser scans [11]. Balling refers to periodic changes in size and shape due to capillary-driven instabilities in the molten pool. These oscillations restrict the resolution of SLM by causing discontinuous tracks, thus causing the formation of voids, increasing the surface roughness, and impeding the formation of extremely precise geometries [11–13]. While keyholing is a result of the recoil pressure generated by the intense vaporization occurring directly beneath the laser at high energy densities.

The achievement of a high-quality part is a significant concern in SLM. The formation of pores in fabricated parts using SLM can have deleterious consequences for the life of fatigue and the ductility of parts [14]. Moreover, poor surface quality could require lengthy and costly post-production finishing processes, which are often performed manually due to the complex shapes of the parts produced, thereby undermining the benefits of utilizing additive manufacturing techniques for industrial production [12]. Hardness is also of significant importance for SLM, as it influences the strength, durability, and dimensional stability of fabricated parts. Lower hardness generally results in lower resistance to deformation, wear, and fatigue.

Hence, understanding the impact of various 3D printing parameters on the process outcome is crucial for the fabrication of high-quality SLM parts, making the SLM process a significant focus of extensive research endeavors. Abdulla et al. [4] found that relative density is significantly impacted by laser power and scanning speed of components: the use of high speed and high power leads to better densification of samples. Kamath et al. [15] found that selecting an excessively high power for a specific speed can cause overheating, resulting in deeper laser penetration and the formation of pores filled with inert gas. Furthermore, Zhang et al. [16] found that an increase in laser power leads to an enlargement of the grain size or pores, whereas an increase in scanning speed results in a reduction of the grain size. According to the Hall-Petch effect, as the grain size of a material decreases, its strength increases. For surface roughness, Sadali et al. [17] examined the relationship between scanning speed and surface roughness. The study found that when the scanning speed increases, the surface roughness exhibits a parabolic pattern, initially increasing and then decreasing. Delgado et al. [18], Kempen et al. [19], and Song et al. [20] studied the relationship between hardness and scan speed and found that hardness and scan speed are inversely related. Significant benefits and enhancements can be realized during the planning of the SLM production process by predicting surface roughness [12], relative density and hardness in advance using process parameters.

Modeling of AM processes is essential for estimating part performance characteristics and optimizing process parameters. Adjusting the process parameters can improve different properties of the fabricated parts, such as relative density, surface roughness, and hardness. This can be done by using several data-driven methods, such as empirical, experimental, statistical, and machine learning algorithms, to model various AM processes and optimize them. Machine learning algorithms offer opportunities to accurately analyze performance characteristics such as relative density (RD), surface roughness, and hardness of 3D printed parts to improve SLM processes due to the non-linearity and complexity of AM.

Significant research has been conducted to evaluate the potential application of advanced ML processes to predict various product properties and improve the quality of AM outcomes [21–24]. Studies have focused on improving SLM processes to improve the quality of final products [25]. There are many ML algorithms, including genetic algorithms, support vector machines (SVMs), and artificial neural networks (ANN) that effectively model complex systems. In applications associated with materials design, kernel ridge regression (KRR) and support vector regression (SVR) have shown promising results.

Different ML algorithms have been applied to model and predict the relative density or porosity of SLM-fabricated parts. Barrionuevo et al. [26] used decision trees, SVR, gradient booster, multilayer perceptron (MLP) and the Gaussian process to predict RD of 316L fabricated parts, and the results revealed that MLP achieved the highest accuracy based on the coefficient of determination (R^2^) and the root mean square error (RMSE). Abdulla et al. [3,4] compared and evaluated the performance of KRR, ridge regression and SVR in predicting RD of 316L stainless steel samples and found that KRR generated the highest accuracy in terms of R^2^ and mean square error (MSE). Gor et al. [27] implemented K-nearest neighbour (KNN), ANN, linear regression, and SVM to create a density prediction model. The study by Gor et al. [27] revealed that ANN outperformed other models in predicting the relative density of 316L stainless steel build parts. XGBoost (a gradient-boosting decision tree), SVR and ANN were developed by Zou et al. [28] to analyze the relative density of Ti-6Al-4V parts. The study found that the optimized XGBoost has a better performance indicator based on R^2^, RMSE, and mean absolute error (MAE).

ML has also been used to predict the surface roughness and hardness of parts fabricated using SLM. Zhang et al. [29] investigated the roughness of the upper surface of 316L stainless steel using backpropagation neural networks (BPNN) with one output and three input parameters. The high-precision accuracy provided by the BPNN model can be leveraged in SLM process optimization. Soler et al. [30] developed an ANN algorithm to predict surface roughness of Ti6Al4V fabricated parts using SLM. Gogulamudi et al. [31], used ANN with a feedforward backpropagation algorithm to predict the surface roughness and hardness of AlSi10Mg parts printed using SLM.

ML methods such as ANN and SVM have shown great promise in accurately predicting surface roughness and relative density, or porosity, of the 3D printed metals [27]. However, research on the application of ML methods to predict surface hardness of 3D printed metallic materials and the amount of data available to develop such models is limited. Particularly, there is little research that simultaneously attempted to predict relative density, surface roughness, and hardness of 3D printed AlSi10Mg samples, with respect to SLM printing parameters, using ML methods.

The processing of SLM Al-Si alloys, especially AlSi10Mg, has received more attention recently due to their promising lightweight applications [32]. AlSi10Mg alloy has been widely used in aerospace, automotive, and domestic industries due to its low density, high strength, and corrosion resistance [33–34]. Several studies have focused on optimizing the process parameters of AlSi10Mg to overcome the limitations inherent to the SLM process. Aboulkhair et al. [35] highlighted the challenges associated with part density and surface roughness and emphasized the importance of adjusting laser power and scanning speed to improve the quality of SLM-processed parts. Maamoun et al. [36] further examined the effects of energy density and powder characteristics on the mechanical properties and surface finish of AlSi10Mg parts, showing that careful tuning of these parameters can enhance part quality and reduce the need for post-processing. However, the AlSi10Mg fabricated parts still do not consistently meet the stringent requirements for industrial applications due to the aforementioned drawbacks of SLM [32].

The primary challenge lies in the inability to reliably predict and optimize the key quality parameters—relative density, surface roughness, and hardness—of AlSi10Mg parts produced by SLM. Current approaches have not extensively addressed the non-linear interactions between process parameters that influence these properties, leaving a gap in achieving the necessary part qualities.

Therefore, this study will further contribute to the literature to address these demerits by constructing a comprehensive data set from the literature and laboratory experiments to evaluate the possibilities of using machine learning algorithms to predict the relative density, surface roughness and hardness of AlSi10Mg fabricated parts. The ML algorithms that are considered in this study are SVR, KRR, random forest (RF), Lasso regression, and ANN. The SLM process parameters considered in this study are the hatch distance, layer thickness, laser power, and speed. Based on leave-one-out cross-validation (LOOCV), the models are evaluated by the MSE and R^2^ between the predicted and actual values.

The following sections of the paper are organized as follows. Section 2 presents the methodology used to compile the data set from sources in the literature, describes the experimental procedure used to measure relative density, roughness of the surface, and hardness, and provides an explanation of the machine learning models employed in the study. Subsequently, Section 3 presents the analytical and numerical results obtained and discusses the notable findings. Finally, Section 4 offers the concluding remarks.

## 2. Methodology

**Fig 1:**
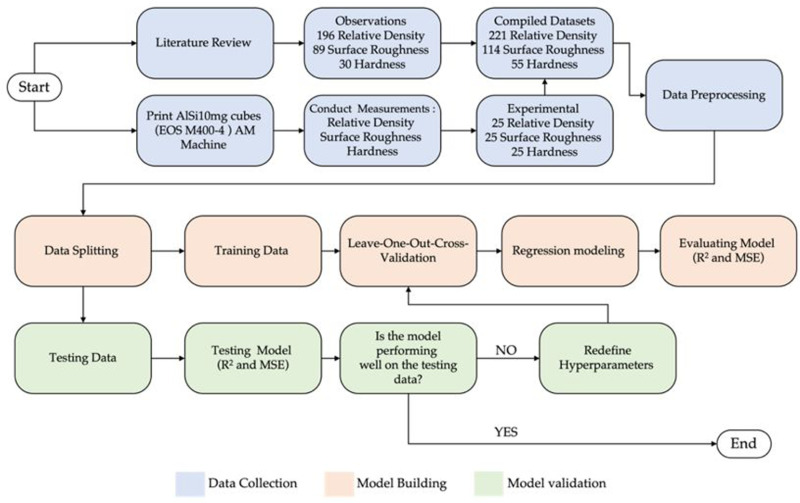
Schematic representation of the methodology used to evaluate the ML algorithms for predicting the relative density, surface roughness, and hardness of AlSi10Mg parts produced using SLM.

The methodology used in this study to predict the relative density, surface roughness, and hardness of the fabricated samples can be summarized in three steps: data collection, model building, and model validation, as illustrated in Fig 1. In the data collection phase, the AlSi10Mg data were compiled from both data from the literature and the experimental data collected for this study. The data was then cleaned and split into training and testing sets. The algorithms used in this study were trained using Leave-One-Out Cross-Validation (LOOCV) and subsequently evaluated. Finally, these models were tested on the test data set to assess their performance.

### 2.1. Material

**Fig 2:**
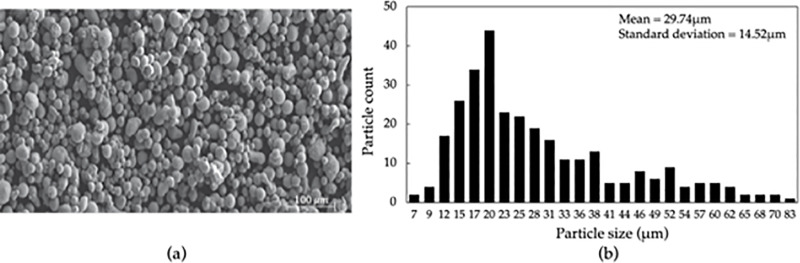
(a) SEM image of EOS AlSi10Mg powder, and (b) Gaussian distribution of the powder particle size showing mean and standard deviation [37].

In this study, a commercial grade EOS AlSi10Mg powder with a spherical shape and particle size distribution of 25-70 μm was used to produce specimens using SLM. Fig 2(a), and 2(b) shows a scanning electron microscopy (SEM) image of the AlSi10Mg powder, and Gaussian curve of the particle size distribution respectively. The chemical composition of the powder is shown in Table 1.

**Table 1:** Chemical composition of AlSi10Mg [37].

Element	Fe	Cu	Mn	Ni	Zn	Pb	Sn	Ti	Mg	Si	Al
Composition (wt%)	0.55	0.05	0.45	0.05	0.10	0.05	0.05	0.15	0.25–0.45	9.0–11.0	Balance

### 2.2. Data collection from literature

The data used in this research were obtained from both sources from the literature and experimental measurements, focusing on the relative density, surface roughness, and hardness of AlSi10Mg samples fabricated using selective laser melting (SLM). These properties were examined under various process parameters, including laser power, hatch distance, scan speed, and layer thickness. The experimental conditions from the literature are detailed in Tables 2–4 for relative density, surface roughness, and hardness, respectively. The compiled datasets for these properties are summarized in S1, S2, and S3 Tables in the supplementary materials.

**Table 2:** Experimental conditions for relative density measurements.

Authors	Experimental Conditions			
	Machine	Powder	Fabricated parts	Density/Porosity Measurement Method
Maamoun et al. [38]	EOSINT M290 machine: Equipped with 400 W Yb-fiber laser. Laser beam diameter of 100 m. Oxygen content below 0.1%.	Gas-atomized AlSi10Mg powder with particle size distribution of 12–110 μm.	Cubes of size 15 × 15 × 15 mm3.	Archimedes method.
Han et al. [39]	Self-developed machine (LSNF-I): Equipped with a wave IPG YLR-200 fiber laser. Maximum laser power of 200 W. Foal diameter of 100 μm.	Gas-atomized AlSi10Mg powder with particles size in distribution of 18–50 μm and spherical shape.	Oblique Rectangular prism.	–
Pei et al. [40]	Self-developed SLM 150: Build chamber of 150 × 150 × 150 mm3. Random rotation scan strategy.	Gas-atomized AlSi10Mg powder with spherical shape and average particle diameter of 26.53 μm.	Cubic samples of size 10 × 10 × 6 mm3.	Archimedes method.
Sing et al. [41]	SLM250 HL machine: Equipped with a Gaussian beam fiber laser of power up to 400 W. - Focal diameter of 80 μm.	AlSi10Mg powder with particle size distribution of 20–63 μm.	Hollow cylinder of 15 mm diameter.	Archimedes method.
Read et al. [34]	Concept Laser M2 Cusing machine: Yb-Fiber laser. Laser power up to 200 W. Laser track width of 150 μm. Laser scan speed up to 7000 mm/s. Argon atmosphere with an oxygen-content < 0.1%. Island scanning strategy.	AlSi10Mg powder with particle size distribution of 20–63 μm.	Cubes of size 10 × 10 × 10 mm3.	Samples were analysed using a Zeiss Axioskop microscope, an Axioskop 2 image analyser and AxioVision software after polishing.
Kempen et al. [42]	Concept Laser M1 SLM machine: Fiber laser power of 200 W. Laser beam diameter of about 150 μm. Island scanning strategy.	AlSi10Mg powder with an average particle size of 16.3 μm.	–	Archimedes method.
Aboulkhair et al. [43]	Realizer GmbH SLM-50 machine: Equipped with a 100 W yttrium fiber laser (YLM-100-AC). Argon atmosphere with an oxygen level below 0.5%. X, 2X, Alternating, Pre-sinter, X&Y 2HS and Overlap scanning strategies used.	AlSi10Mg powder with an average particle size of 50 μm.	Cubes of size 5 × 5 × 5 mm3.	ImageJ software is used to scan electron microscope images.
Yap et al. [44]	SLM 250 HL machine	AlSi10Mg powder.	–	Archimedes method.
Raus et al. [45]	SLM 125 HL machine: Equipped with a 400 W fiber laser. 80 μm laser beam spot. Build chamber of 125 × 125 × 125 mm3. Stripe scanning strategy.	AlSi10Mg powder with particles size ranges from 5 to 50 μm.	Cubes of size 10 × 10 × 10 mm3.	Archimedes method.
Kan. et al. [46]	Commercial LPBF system: Rotation scan strategy.	AlSi10Mg with particles size ranges from 15.6 to 48.3 μm.	Blocks of size 90 × 90 × 20 mm3.	Scanning electron microscope images.
Bai et al. [47]	Concept Laser X Line 1000 machine.	Gas-atomized AlSi10Mg powder with particle size distribution ranging between 20 and 60 μm and spherical shape.	Cubes of size 10 × 10 × 10 mm3.	Archimedes method.
Wang et al. [48]	Renishaw AM-250 powder- bed machine: Maximum power of 400 W.	Hypoeutectic AlSi10Mg. with particle size distribution range between 5 and 50 μm.	Cubes of size 10 × 10 × 10 mm3.	Densitometer (Beyond, DE-120M).

**Table 3:** Experimental conditions for the surface roughness measurements.

Authors	Experimental Conditions			
	Machine	Powder	Fabricated parts	Surface Roughness Measurement Method
Han et al. [39]	Self-developed machine (LSNF-I): Equipped with a wave IPG YLR-200 fiber laser. Laser power of 200 W. Foal diameter of 100 μm.	Gas-atomized AlSi10Mg powder with particles size in distribution of 18–50 μm and spherical shape.	Oblique Rectangular prism.	MATLAB-based shadow measurements of optical microscope images.
Pei et al. [40]	Self-developed SLM 150 equipment: Build chamber of 150 × 150 × 150 mm3. Random rotation scan strategy.	Gas-atomized AlSi10Mg powder with spherical shape and average particle diameter of 26.53 μm.	Cubic samples of size 10 × 10 × 6 mm3.	Laser Scanning Confocal Microscope (LEXT OLS4100, Japan).
Kempen et al. [42]	Concept Laser M1 SLM machine: Fiber laser power of 200 W. Laser beam diameter of 150 μm. Island scanning strategy.	AlSi10Mg powder with an average particle size of 16.3 μm.	–	Contact surface profilometer, Talysurf 120L. 3D profiles are composed by taking 21 successive 2D roughness measurements of 10mm, with 100μm in between these 2D measuring lines.
Poncelet et al. [49]	ProX DMP 200 machine: Laser operating at 1075 nm wavelength. Spot size of 70 μm and 273.6 W maximum power. Argon gas atmosphere, with a residual level of O2 concentration smaller than 500 ppm.	AlSi10Mg gas atomized spherical powder with an average diameter of 15–53 μm.	Cubic samples of size 10 × 10 × 10 mm3.	White light interferometry with an MSA-500 Micro System Analyzer (Polytec) built on a vibration-isolation table (SUSS, VIT80x range).

**Table 4:** Experimental conditions for the hardness measurements.

Authors	Experimental Conditions			
	Machine	Powder	Fabricated parts	Hardness Measurement Method
Maamoun et al. [38]	EOSINT M290 machine: Equipped with 400 W Yb-fiber laser. Laser beam diameter of 100 m. Oxygen content below 0.1%.	Gas-atomized AlSi10Mg powder with particle size distribution of 12–110 μm.	Cubes of size 15 × 15 × 15 mm3.	Automatic Clemex CMT tester. measured value was an average of 5–10 indentations made by a 200 gf load applied over a 10 s dwell time along the tested area.
Poncelet et al. [49]	ProX DMP 200 machine: Laser operating at 1075 nm wavelength. Spot size of 70 μm and 273.6 W maximum power. Argon gas atmosphere, with a residual level of O2 concentration smaller than 500 ppm.	AlSi10Mg gas atomized spherical powder with an average diameter of 15–53 μm.	Cubic samples of size 10 × 10 × 10 mm3.	DuraScan G5, a Vickers tip indenting with 200 g (HV 0.2). Ten indents were performed per measurement.
Yusuf et al. [50]	Concept Laser M2 SLM machine: Chamber with N2 gas environment at room temperature. Alternating bidirectional scan strategy.	AlSi10Mg gas atomized powder.	200 mm long cylindrical rods with a diameter of 10 mm.	Future Tech FM-300 Vickers hardness testing machine under an applied load of 500 gf with a dwell time of 15 s.
Tridello et al. [51]	SLM Solutions 500 HL quad 4 × 400 W machine.	AlSi10Mg gas atomized powder.	Cubic samples.	Ultrasonic VHCF tests.
Mfusi et al. [52]	SLM Solutions M280 machine.	AlSi10Mg powder.	Tensile samples.	Zwick Micro/Macro Vickers hardness tester with a load of 300 g and 20 indents.

#### 2.2.1 Selection criteria for literature data.

The selection of sources from the literature followed specific criteria to ensure both the relevance and quality of the data set.

##### Relevance

: Only studies directly addressing SLM of AlSi10Mg with detailed experimental data were included. These studies focused on the effects of key process parameters on material properties.

##### Experimental rigor

: Priority was given to studies that provided comprehensive descriptions of their experimental setups, including the types of SLM machines used, the process parameters, and methods for measuring relative density, surface roughness, and hardness.

##### Data completeness

: The selected studies offered complete data sets that encompass all necessary process parameters and the corresponding material property measurements. The specific experimental conditions for each study are presented in Tables 2, 3, and 4, with the data compiled summarized in S1, S2, and S3 Tables in the supplementary materials.

#### 2.2.2 Evaluation of data quality and handling of bias.

To ensure high data quality and mitigate potential biases, the following steps were implemented:

1. **Cross-Verification**: Data points were cross-referenced between multiple studies to identify and exclude any outliers or inconsistencies, minimizing the impact of methodological differences.
2. **Normalization**: Differences in SLM machine specifications, powder characteristics, and measurement techniques were addressed by normalizing the data, ensuring consistent and accurate model training across sources.
3. **Bias Identification**: Potential biases, such as machine setting variations or powder properties, were carefully identified. Data from studies with significantly different powder particle sizes or laser power ranges were either weighted accordingly or excluded to avoid undue influence on the results.

#### 2.2.3 Integration with experimental data.

To complement the data in the literature, experimental measurements were performed on AlSi10Mg samples fabricated using the EOS M400-4 SLM machine. These measurements extended the range of process parameters beyond those explored in the literature, resulting in a more comprehensive data set. The combined data, detailed in S1, S2, and S3 Tables in the supplementary materials, enhanced the predictive power of the machine learning models employed in this study, leading to more robust and accurate predictions.

### 2.3. Experimental procedure

#### 2.3.1. *Design of experiments (DOE) .*

To design the experimental scheme, a design of experiments (DOE) using a Response Surface Methodology (RSM) is used. RSM’s most widely used technique, Central Composite Design (CCD) [53], was implemented. The factors used in this experiment are laser power, scanning speed, hatch distance, and layer thickness. Each input factor corresponded to five different levels with intervals of (–2, 2) in DOE. The maximum and minimum levels for each factor were specified based on the ranges of data from the literature and the capabilities of the machine.

**Fig 3:**
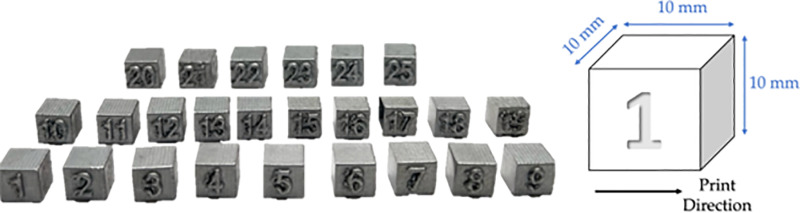
AlSi10Mg cube samples with the dimensions and printing direction fabricated using the EOS M400-4 AM machine.

Specimens with cubical dimensions of 10 ×  10 ×  10 mm^3^ as shown in Fig 3 were fabricated using laser powder-bed fusion (LPBF) technique with an EOS M400-4 AM machine [54]. The architecture of this machine is an open source allowing to print with various sets of process parameters, such as laser power, hatch distance, scan speed, and layer thickness. The machine is equipped with four 400 W fiber lasers, with a maximum scan speed up to 7000 mm/s and has a 400 ×  400 ×  400 mm total build volume. The build plate was preheated to a temperature between 145 and 165°C prior and during the printing process. One sample was printed for each process parameter set, resulting in 25 printed cubic samples.

#### 2.3.2. Density measurement.

The densities of the samples were measured using the Archimedes method by weighing the samples in air and the amount of water displaced using the AS 220.R2 Analytical Balance weighing chamber. Relative density values are defined as the ratio between the measured density of each of the printed samples and the bulk density of the AlSi10Mg alloy (e.g., 2.65 g/cm^3^).

#### 2.3.3. Surface roughness measurement.

**Fig 4:**
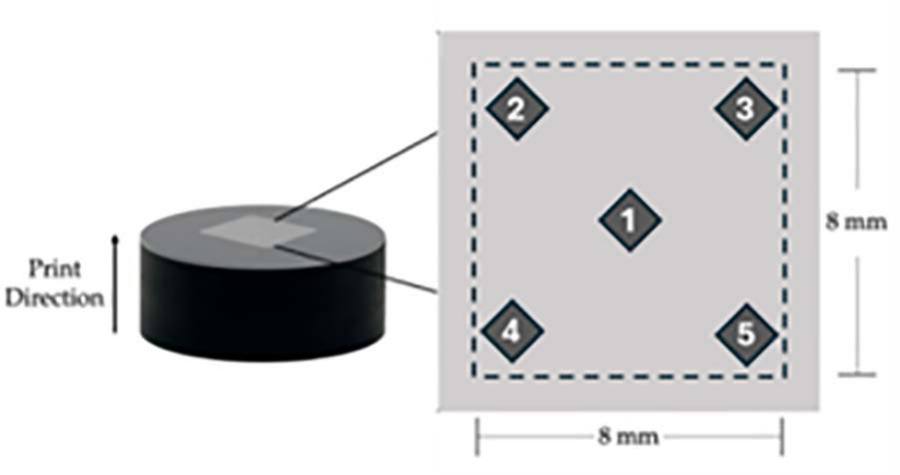
Mounted sample used for surface roughness and hardness measurements at five locations.

Roughness analyzes of as-printed samples were performed with the Alicona InfiniteFocusG5 [55] machine. Measurements were taken in the X-Y plane with a 5x objective lens to test the top surface with an area of approximately 8 ×  8 mm^2^ as illustrated in **Fig 4**. The cutoff wavelength used for each profile analyzed was aligned with the EN ISO 116610 and ISO 4288 standards.

#### 2.3.4. Hardness measurement.

After the surface roughness measurements, the microhardness of the AlSi10Mg samples were measured using a Vickers hardness test (HV 5.0) test with a load of 5.0 kg. Measurements were taken according to ASTM E384-17 [56] using a Struers/ Emco-Test Duramiin-A2500 machine. First, samples were sandblasted to remove residual powder and then mounted using a thermosetting resin with a Struers Prontopress-10 machine. Next step involved grinding and polishing with a Terapol-21 machine, beginning with coarse and fine grinding followed by rough and fine polishing using diamond abrasives at various loads and speeds, with details as specified in Table 5 below. Lastly, for the microstructural characterization, samples were etched for thirteen seconds with Keller’s reagent, consisting of a mixture of hydrofluoric, hydrochloric, and nitric acids diluted in water, applied via swab for detailed surface analysis.

**Table 5:** Details of the grinding and polishing steps used.

Process	Abrasive	Lubricant	Force	Speed	Time
Coarse grinding	Diamond 60 μm	Water	25 N	300 rpm	Until plane
Fine grinding	Diamond 9 μm	Largo 9	30 N	150 rpm	4 min
Coarse polishing	Diamond 3 μm	DiaPro Mol R3	25 N	150 rpm	3 min
Smooth polishing	Colloidal silica 0.04 μm	OP - U non-dry	15 N	150 rpm	1 min

### 2.4. Data pre-processing

The data from the literature and experiments contained 221 observations for relative density, 114 observations for surface roughness, and 55 observations for hardness. The data cleansing process began by identifying potential outliers based on the least squares regression’s standardized residuals. Standardized residuals should fall between -2 and 2, and any observation with a standard residual greater than 3 in absolute value is considered an outlier and is removed [57]. After the removal of outliers, the data sets were reduced to 218, 110, and 52 observations, for relative density, surface roughness, and hardness, respectively, suitable for data analysis and modeling. The data covariates were then normalized to have a mean of zero and a unit standard deviation. The size of the surface roughness and hardness data sets constructed in this study is approximately six times and three times larger, respectively, than the data used in [31] (that is, 20 points), highlighting the reliability of this study.

### 2.5. Machine learning algorithms

In this study, we use five distinct machine learning algorithms to model the relationships between the selective laser melting (SLM) process parameters and the resulting properties of AlSi10Mg samples, such as surface roughness, relative density, and hardness. The selected algorithms—Artificial Neural Networks (ANN), Support Vector Regression (SVR), Kernel Ridge Regression (KRR), Random Forest (RF) and Lasso Regression—were chosen for their complementary strengths, allowing for a comprehensive comparative analysis of predictive modeling techniques in the context of SLM.

#### 2.5.1. Lasso regression.

Lasso Regression, or Least Absolute Shrinkage and Selection Operator, was included for its ability to perform variable selection and regularization, thus improving the model’s simplicity and interpretability. Lasso helps identify the most significant process parameters by shrinking the coefficients of less important variables to zero. Lasso effectively reduces model complexity and avoids overfitting, which is crucial when working with data sets with correlated features [58].

Lasso uses L_1_ regularization to penalize and shrink the coefficients of the less important variables toward zero, resulting in a model with only the most important features selected [58]. The regularization term is a penalty term commonly denoted by lambda (λ≥0 ), which is multiplied by the absolute value of the coefficient. The Lasso loss function can be mathematically expressed as shown in Equation (1) [58]:


$$L\left(\beta \right)={\left(y-X\beta \right)}^{2}+\lambda \overset{p}{\underset{i=1}{\sum }}\left|\beta_{i}\right|$$ (1)

where *λ*. is the regularization (penalty) term, *X* is an n×p matrix, and *y* is the outcome vector.

#### 2.5.2. Kernel ridge regression (KRR).

Ridge Regression (RR) addresses the problem of multicollinearity of linear models of the form y=Xβ+ϵ through the addition of the regularization parameter λ≥0 to the loss function [59], as expressed in Equation (2):


$$L(\beta )=\frac{1}{2}{\left(y-X\beta \right)}^{T}(y-X\beta )+\frac{\lambda }{2}||\beta^{T}\beta |{|}^{2}$$ (2)

The solution to (2) is given by $\hat{\beta }={\left(X^{T}X+\lambda I_{d}\right)}^{-1}X^{T}y,$ where I is a d×d identity matrix. The coefficient vector *β* can be expressed as a linear combination of the data points such that $\beta =X^{T}\alpha$ [4]. This leads to Equation (3):


$$y=XX^{T}\alpha +\epsilon =G\alpha +\epsilon$$ (3)

where the matrix $G=XX^{T}$ is a Gram matrix.

Kernel Ridge Regression (KRR) addresses the non-linearity in data by applying a mapping function $\phi \left(.\right)$ that maps the data to a higher dimensional space. The Kernel Ridge approach was specifically chosen to handle potential multicollinearity among the process parameters while maintaining predictive accuracy. The kernel function uses the dot products such that $K=k\left(x_{i},x_{j}\right)$ =  $\phi \left(x_{i}\right),\phi (x_{j})$ [59,60]. The most used kernels are the linear, polynomial, and radial basis function (RBF) kernels. For this study, we use the RBF kernel, which is given by Equation (4):


$$k\left(x_{i},x_{j}\right)=e^{-\gamma |\left|x_{i}-x_{j}\right|{\left.\right|}^{2}}$$ (4)

where γ>0 is the kernel width.

Replacing the matrix *G* in (3) with the kernel *K*, the kernel ridge regression model is then given by Equation (5):


y=Kα+ϵ (5)

The KRR function to be minimized with regard to the dual variable *α* becomes as expressed in Equation (6):


$$f\left(\alpha \right)=\frac{1}{2}{\left(y-K\alpha \right)}^{T}\left(y-K\alpha \right)+\frac{\lambda }{2}{\left|\left|\alpha \right|\right|}^{2}$$ (6)

to which the solution is provided by Equation (7), as follows [4]:


$$\alpha ={\left(K+\lambda I_{N}\right)}^{-1}y$$ (7)

Now, if the matrix ($K+\lambda I_{N}$) is dense, iterative methods would be optimal [59].

#### 2.5.3. Support vector regression (SVR).

Support Vector Regression (SVR), like KRR, was selected due to its strong performance in high-dimensional space and its ability to handle nonlinear relationships using kernel functions. This algorithm works based on the structural risk minimization principle and is known for its lower sensitivity to dimensionality and its ability to achieve a lower generalization error of the regression model [61,62]. SVR finds the best possible hyperplane that passes through the maximum number of data points and provides flexibility in defining the acceptable error level in the model. The objective of SVR is to find a fitting function $f\left(x\right)=\left\{w,x\right\}+b$, where *w* is the weight vector, and *b* is the constant (bias), that can predict the target value $\left(y_{i}\right)$ for a given training data point $\left(x_{i}\right)$ with a deviation smaller than a defined value ε [26].

#### 2.5.4. Random forest (RF).

Random Forest is an ensemble method involving the training of multiple decision trees and the selection of the ensemble tree class with the best overall performance [63]. RF was chosen for its ability to handle large datasets with higher dimensionality and for providing insights into the importance of features, which helps to understand the influence of each process parameter on the resulting material properties. The method involves building a set of n decision trees also known as estimators $\left\{R_{1}\left(X\right),R_{2}\left(X\right),....,R_{N}\left(X\right)\right\}$, where $X=\left\{x_{1},x_{2},...,x_{p}\right\}$, is a p-dimensional input vector. The ensemble generates N outputs corresponded to each tree $\hat{Y}=R_{1}\left(X\right),R_{2}\left(X\right),....,R_{N}\left(X\right)$, where each tree’s output is represented by ${\hat{Y}}_{n}$, n=1,...,N. RF takes the average to control overfitting and improve the predictive accuracy [26].

#### 2.5.5. Artificial neural networks (ANN).

**Fig 5:**
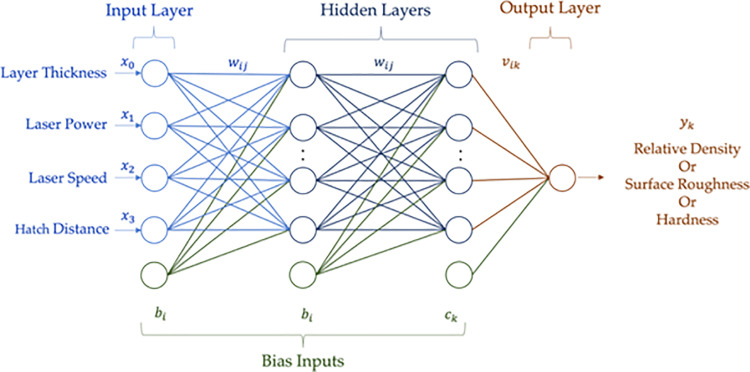
Schematic of the ANN model.

ANN is a powerful alternative to conventional techniques based on neuronal structure. ANN consists of interconnected elements called neurons. In this study, ANN was selected because it can capture many types of relationships, and it applies to problems where the relationships between the variables might be non-linear or quite dynamic [63]. Multi-layer Perceptron (MLP) is the most used neural network that can model complex functions and is robust to irrelevant input and noise [63]. As shown in Fig 5 MLP consists of an input layer, one or more hidden layers, and an output layer. The neurons of the layers are fully connected to the neurons of the previous and next layers. Each neuron’s output is calculated by applying an activation function to a weighted sum of its inputs. In this study, rectified linear unit function (RELU) is used for all layers as an activation function.

### 2.6. Model evaluation with leave-one-cross-validation

In this study, both Leave-One-Out Cross-Validation (LOOCV) and 10-fold cross-validation were used to validate machine learning models. LOOCV is particularly useful when the data set size is small, as it maximises the use of each data point by using it once for validation and the rest for training. Although LOOCV can be computationally intensive, it provides low-bias estimates, which is crucial for smaller datasets, such as the one used in this study [64]. The models were evaluated using both R² (coefficient of determination) and Mean Squared Error (MSE) as performance metrics, providing a balance between measuring goodness of fit and prediction error. Both metrics were calculated for the results obtained from LOOCV and the 10-fold cross-validation, as provided in Table 7.

**Table 7:** Optimal tuning parameters and accuracy of the relative density, surface roughness and hardness models.

Model	Parameters	$R^{2}$	MSE
Relative density models using LOOCV			
ANN	α = 0.01	0.772	0.232
SVR	γ = 2, C=35	0.745	0.255
KRR	γ = 2, α = 0.01	0.689	0.311
RF	n-estimators = 100	0.634	0.367
Lasso	*λ* = 0.12	0.198	0.802
Relative density models using 10-fold CV			
ANN	α = 0.01	0.563	0.438
SVR	γ = 0.45, C=100	0.522	0.479
KRR	γ = 0.1, α = 0.1	0.484	0.516
RF	n-estimators = 10	0.455	0.544
Lasso	*λ* = 0.1	0.290	0.710
Surface roughness models using LOOCV			
ANN	α= 0.01	0.677	0.395
SVR	γ = 0.1, C=15	0.326	0.673
KRR	γ=0.01, α = 0.01	0.398	0.601
RF	n-estimators = 100	0.452	0.536
Lasso	*λ* = 0.1	0.218	0.782
Surface roughness models using			
ANN	α= 0.01	0.624	0.375
SVR	γ = 0.1, C=10	0.311	0.688
KRR	γ=1, α = 0.1	0.310	0.690
RF	n-estimators = 100	0.370	0.629
Lasso	*λ* = 0.01	0.228	0.771
Hardness models using LOOCV			
ANN	*λ* = 0.01	0.704	0.122
SVR	γ = 1, C=5	0.629	0.371
KRR	γ = 0.5, α = 0.01	0.535	0.465
RF	n-estimators = 100	0.488	0.512
Lasso	*λ* = 0.1	0.144	0.856
Hardness models using 10-fold CV			
ANN	*λ* = 0.01	0.698	0.305
SVR	γ = 1, C=100	0.546	0.456
KRR	γ = 0.005, α = 0.001	0.512	0.488
RF	n-estimators = 100	0.490	0.510
Lasso	*λ* = 1	0.181	0.819

The data for relative density and surface roughness are divided into 80% training, and 20% testing and the LOOCV was applied on the training. For the hardness data set, LOOCV was applied to the entire data set due to the limited number of observations. Using a MacBook Pro with 16 GB of DDR3 RAM, the five regression models utilized in this study to predict the relative density, surface roughness, and hardness are executed using Scikit-learn and TensorFlow. Furthermore, grid search was performed to find the optimal parameters for all the ML algorithms used.

## 3. Results and discussion

### 3.1. Experimental results

**Fig 6:**
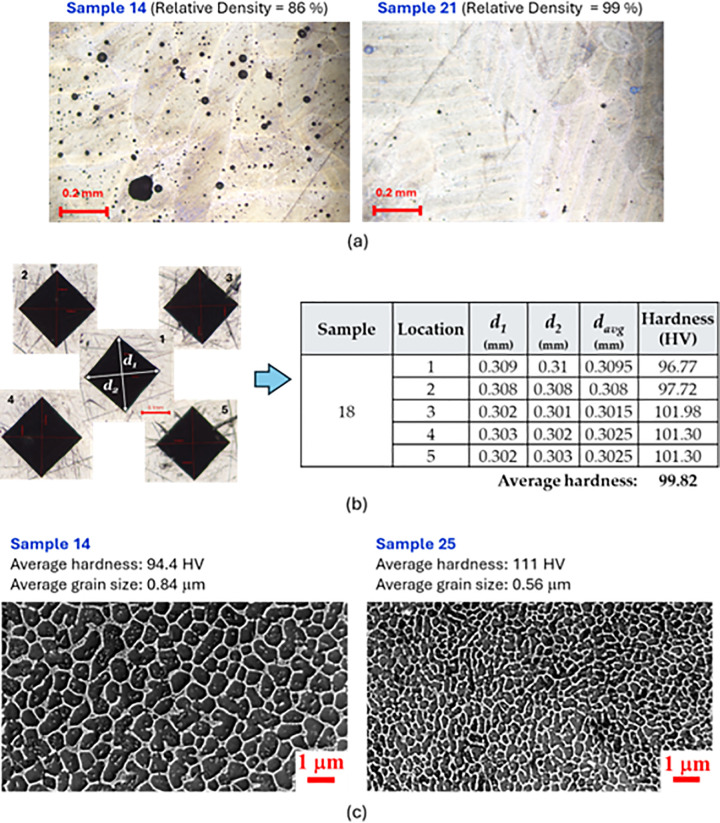
(a) Optical microscope images showing porosities in sample 14 and 21, (b) mounted sample with location for hardness measurements and detailed measurements of sample 18, (c) SEM images of sample 14 and 25 showing the grain size distribution.

The relative density measurement results for all 3D printed samples are given in Table 6. Some of the samples measure near to 100% relative density, which is attributed to the measuring method used and are denoted a relative density of less than 100% (e.g., < 100%). In order to verify the relative density measurements in Table 6, several of the printed samples were polished and examined with optical microscope. The micrographs, as depicted in Fig 6(a), clearly show the appearance of porosities agreeing well with the measurements of relative densities presented in Table 6. For example, Fig 6(a) for sample 14 shows large number of porosities confirming the measured low relative density of 86%, while sample 21 shows very small number of porosities attributed to its high relative density near to 100%.

**Table 6:** Selected process parameters and measured relative density, surface roughness, hardness for the 3D printed samples.

Sample No.	Process Parameters				Measured Values			
	Laser Power (W)	Scan Speed (mm/s)	Hatch Distance (mm)	Layer Thickness (mm)	Relative Density (%)	Surface Roughness Sa (μm)		Hardness (HV)
							Average (HV)	Standard Deviation
1	300	938	0.12	0.03	<100	13.49	108.6	6.58
2	700	938	0.12	0.03	98.9	25.66	94.4	5.03
3	300	2313	0.12	0.03	90.7	11.08	117.0	3.24
4	700	2313	0.12	0.03	97.9	14.91	114.0	4.18
5	300	938	0.31	0.03	<100	18.08	103.4	4.04
6	700	938	0.31	0.03	95.1	16.15	102.2	3.35
7	300	2313	0.31	0.03	88.4	21.61	65.8	3.27
8	700	2313	0.31	0.03	92.2	17.86	81.8	8.64
9	300	938	0.12	0.05	<100	21.98	108.0	1.22
10	700	938	0.12	0.05	91.5	28.57	94.4	9.74
11	300	2313	0.12	0.05	<100	18.35	82.2	9.37
12	700	2313	0.12	0.05	93.3	18.79	95.0	7.45
13	300	938	0.31	0.05	92.9	16.07	107.8	4.82
14	700	938	0.31	0.05	86.8	20.04	94.4	4.51
15	300	2313	0.31	0.05	91.3	46.66	79.6	7.06
16	700	2313	0.31	0.05	95.1	27.48	77.6	18.11
17	100	1625	0.22	0.04	84.8	192.79	87.8	9.09
18	900	1625	0.22	0.04	95.5	16.69	101.4	8.29
19	500	250	0.22	0.04	90.9	106.79	81.4	10.62
20	500	3000	0.22	0.04	86.9	9.16	78.8	7.05
21	500	1625	0.03	0.04	99.1	57.50	109.6	8.44
22	500	1625	0.40	0.04	<100	17.23	88.4	3.05
23	500	1625	0.22	0.02	93.5	14.26	104.6	1.52
24	500	1625	0.22	0.06	92.9	16.19	106.6	1.95
25	500	1625	0.22	0.04	89.2	16.41	111.2	2.28

The hardness values in Table 6 are an average value of five indentations in an area of 8 ×  8 mm^2,^ as shown in Fig 6(b). The mean values and standard deviations of the five indentations for each sample are given in Table 6. To verify the hardness measurements, an optical microscope image of the indentations is taken, as shown in Fig 6(b) , and the diagonals of the indentations are measured, and their average diameter $d_{avg}$ is calculated. The Vickers hardness value is then calculated with the relation HV =  1.854 ×  *F*/ $d_{avg}^{2}$, where the applied load is *F* =  5 kg [65]. Fig 6(b) gives an example for sample 18 of such estimation of the hardness as compared to the values tabulated in Table 6, showing a very good agreement. Scanning electron microscopy (SEM) was used to examine the microstructure of the printed samples, providing insights into the surface morphology and the grain size distribution and structure. This analysis helped explain the variations in hardness measurements by linking them to differences in the material’s microstructure. Fig 6(c) presents SEM images of samples 14 and 25, revealing a clear difference in grain size distribution between the two. This variation is reflected in their distinct hardness values as provided in Table 6. For instance, sample 14, with an average grain size of 0.84 µm, has a hardness of 94.4 HV, while sample 25, with a finer average grain size of 0.56 µm, shows a higher hardness of 111 HV. This relationship aligns with the well-known Hall-Petch effect, where smaller grain sizes lead to increased material hardness [66,67].

**Fig 7:**
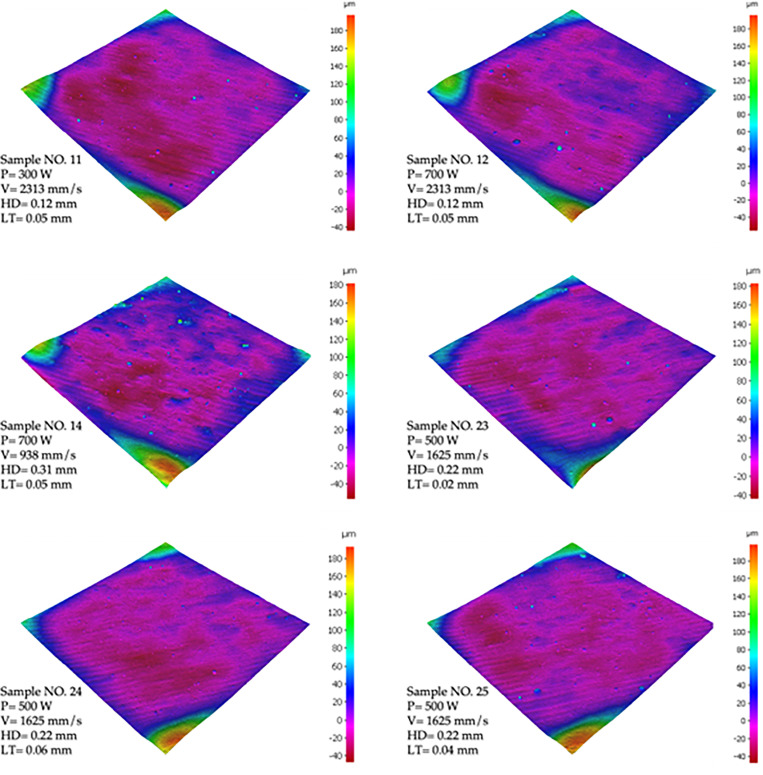
Surface Topology of X-Y plane of SLM-processed AlSi10Mg for six different samples with their corresponding printing parameters.

The arithmetic average roughness (Ra) and the areal average roughness (Sa) were calculated for all samples. However, in this work, Sa was considered as it measures the average height of all measured points on the surface. In contrast, Ra only captures limited information on surface topography variations through a line on the surface. The Sa values, shown in Table 6, are the areal average values derived from the contour surface roughness plots in Fig 7, and are used to characterize surface roughness according to the ISO 25178-2 standard [68]. Furthermore, Fig 7 shows the surface topology of six samples with their corresponding process parameters.

### 3.2. Analysis of data plots

Understanding the relationships between the process parameters and the characteristics of parts produced by SLM is crucial because the properties of such parts depend largely on the input process parameters [69]. Despite representing a portion of the available experimental data on the relative density, surface roughness, and hardness of AlSi10Mg parts fabricated by SLM, the compiled data set could be useful in providing insights of the correlations between the process parameters and the measured properties through observational analysis. S1, S2, and S3 Figs in the supplementary material illustrates how the relative density, surface roughness, and hardness, respectively, change as the laser power, layer thickness, scan speed, hatch distance, and energy density that combines all the four process parameters vary.

### 3.2. Analysis of regression results

**Fig 8:**
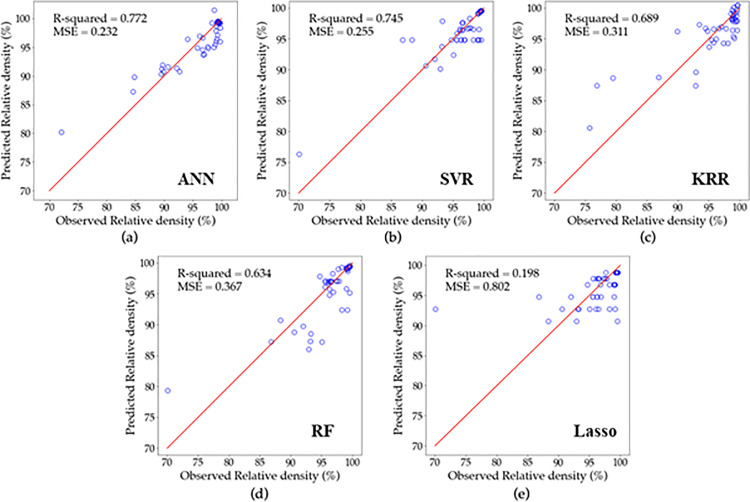
Predicted vs. actual relative density for (a) ANN, (b) SVR, (c) KRR, (d) RF, and (e) Lasso based on LOOCV results.

**Fig 9:**
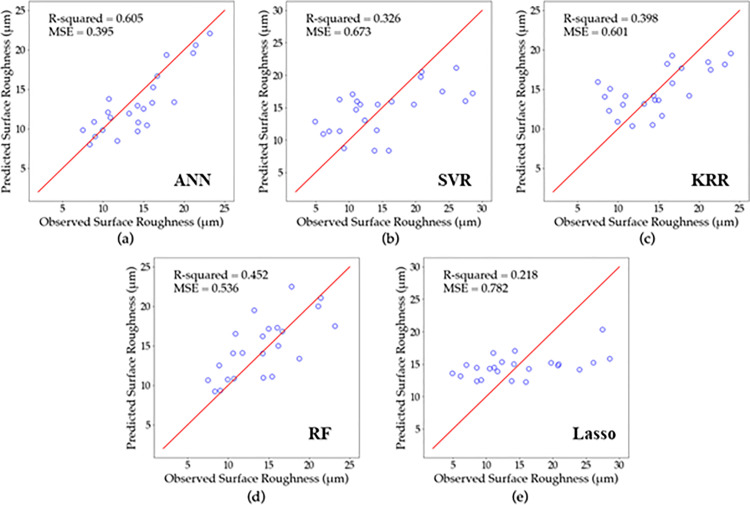
Predicted vs. actual surface roughness for (a) ANN, (b) SVR, (c) KRR, (d) RF, and (e) Lasso based on LOOCV results.

**Fig 10:**
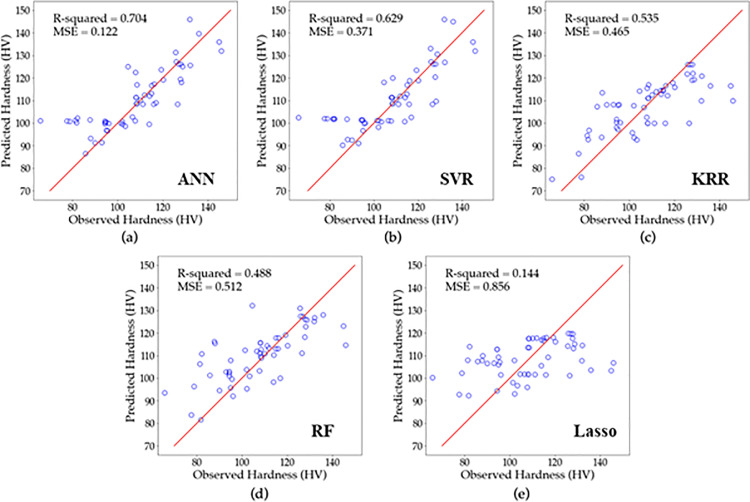
Predicted vs. actual hardness for (a) ANN, (b) SVR, (c) KRR, (d) RF, and (e) Lasso based on LOOCV results.

To assess the effectiveness and suitability of the established models for the real SLM system, Figures 8–10 display a comparison between the experimentally determined relative density, surface roughness, and hardness data and the predicted values, respectively using (a) ANN, (b) SVR, (c) KRR, (d)RF and (e) Lasso regression. If the point lies closer to the diagonal line, this means the predicted value equals the actual value. Furthermore, the results of the relative density, surface roughness, and hardness models are presented in Table 7 along with their optimal tuning parameters and corresponding accuracies as measured by $R^{2}$ and MSE.

For relative density, ANN results presented in Fig 8(a) show the highest accuracy, followed by SVR in Fig 8(b) and KRR in Fig 8(c), as the distribution of their values was closer to the diagonal. In contrast, RF in Fig 8(d) and lasso in Fig 8(e) gave the lowest accuracy, with lasso being the worst. From Table 7, ANN model consisting of two hidden layers with 8 and 16 neurons, was trained for 50 epochs and 4 steps per epoch, achieved an *R*^2^ of 0.772 and *MSE* of 0.232. Similarly, SVR achieved an *R*^2^ of 0.745 and *MSE* of 0.255, with tuning parameters *γ* and *C* of 2 and 35, respectively. The KRR model with tuning parameters of *λ* and *α* equals to 2 and 0.01, respectively resulted in an *R*^2^ of 0.689 and *MSE* of 0.311. Furthermore, RF achieved an *R*^2^ of 0.634 and *MSE* of 0.367, with 100 estimators. The lowest achieved accuracy was by lasso regression model, which had an *R*^2^ of 0.198 and *MSE* of 0.802 using *λ* of 0.12. It is worth noting that both ANN and SVR models have the potential to accurately predict relative density and are adequate for future applications.

The results of the models in predicting surface roughness are presented in Fig 9 and Table 7. The ANN model showed the highest accuracy, with results presented in Fig 9(a). This was followed by the RF model in Fig 9(d) and KRR model in Fig 9(c). The SVR model in Fig 8(b) and Lasso regression model in Fig 9(e) gave the lowest accuracy, as their values were widely dispersed and not along the diagonal. The ANN model with two hidden layers of 8 and 16 neurons, was trained using five 15 epochs and 5 steps per epoch, achieved the highest $R^{2}$ of 0.605 and MSE of 395 as shown in Table 7. The SVR model achieved an $R^{2}$ of 0.452 and MSE. of 0.536, with 100 estimators. The KRR model resulted in an $R^{2}$. of 0.398 and MSE of 0.601, with tuning parameters *λ*. and *α* equals to 0.01, respectively. On the other hand, the SVR model achieved an $R^{2}$ of 0.326 and MSE of 0.673, with tuning parameters γ and C of 0.2 and 25, respectively. Finally, the Lasso regression model achieved the worst accuracy, with an $R^{2}$ of 0.218 and MSE of 0.782 using tuning parameter λ equals to 0.15.

The accuracy of various models in predicting the hardness of a material was examined. Fig 10 displays the results, with the ANN model achieving the highest accuracy Fig 10(a), followed by the SVR in Fig 10(b) and KRR in Fig 10(c) models. The RF in Fig 10(d) and Lasso in Fig 10(e) gave the lowest accuracy. Table 7 provides me details on theerformance of each model. The ANN model with two hidden layers, consisting of 8 and 16 neurons, and 30 epochs achieved the highest $R^{2}$ of 0.704 and MSE of 0.122. The SVR model achieved an $R^{2}$ of 0.629 and MSE of 0.371, with tuning parameters γ and C of 1 and 5, respectively. The KRR model resulted in an $R^{2}$ of 0.535 and MSE of 0.465, with tuning parameters λ and α equals to 0.5 and 0.01, respectively. The RF model achieved an $R^{2}$ of 0.488 and MSE of 0.5, with 100 estimators. The Lasso regression model gave the lowest accuracy, with an $R^{2}$ of 0.144 and MSE of 0.856 using λ of 0.1. Overall, the ANN and SVR models again yielded higher accuracy in predicting the hardness of a AlSi10Mg.

The performance of different models in predicting the mechanical properties of materials, including relative density, surface roughness, and hardness, were examined. ANN and SVR models were consistently outperforming in most cases. On the other hand, Lasso regression models resulted in the lowest accuracy, this is due to the non-linearity in the data. Overall results indicate that ANN was the most effective model due to its ability to deal with problems where the relationships might be non-linear or quite dynamic.

Overall, the results indicate that LOOCV provided better estimates for this small dataset, while 10-fold cross-validation yielded slightly lower accuracy but showed consistency in results across models. All figures are based on LOOCV results to capture the most reliable predictions for the given dataset size.

### 3.3. Printability maps

Identifying the features that have the most significant impact on the target variable is crucial for understanding and optimizing a model’s predictions. In this study, since ANN models are outperforming other models, they were utilized to determine the two most important features for relative density, surface roughness, and hardness. For relative density and hardness, the analysis revealed that scan speed and laser power are the two most influential features. Whereas for surface roughness the two most important features are scan speed and layer thickness.

**Fig 11:**
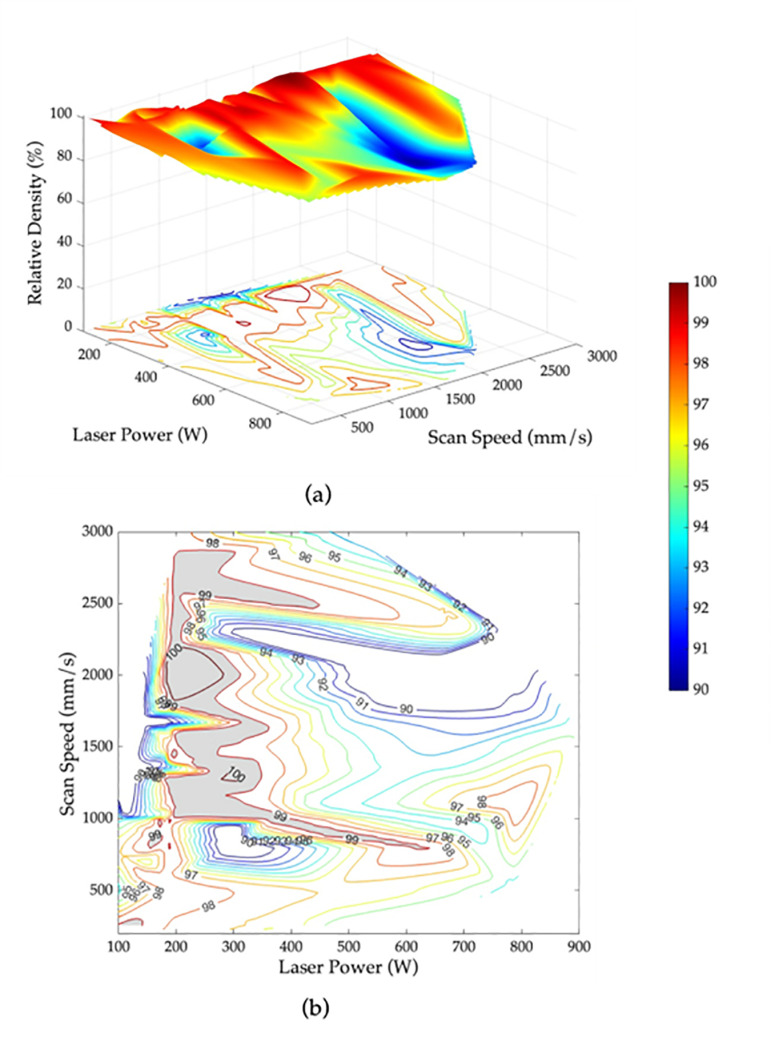
Relative density as a function of laser power and scan speed for AlSi10Mg parts using SLM (a) 3D contour map and (b) 2D contour map with the optimized processing window shown in grey for relative density >  99%.

**Fig 12:**
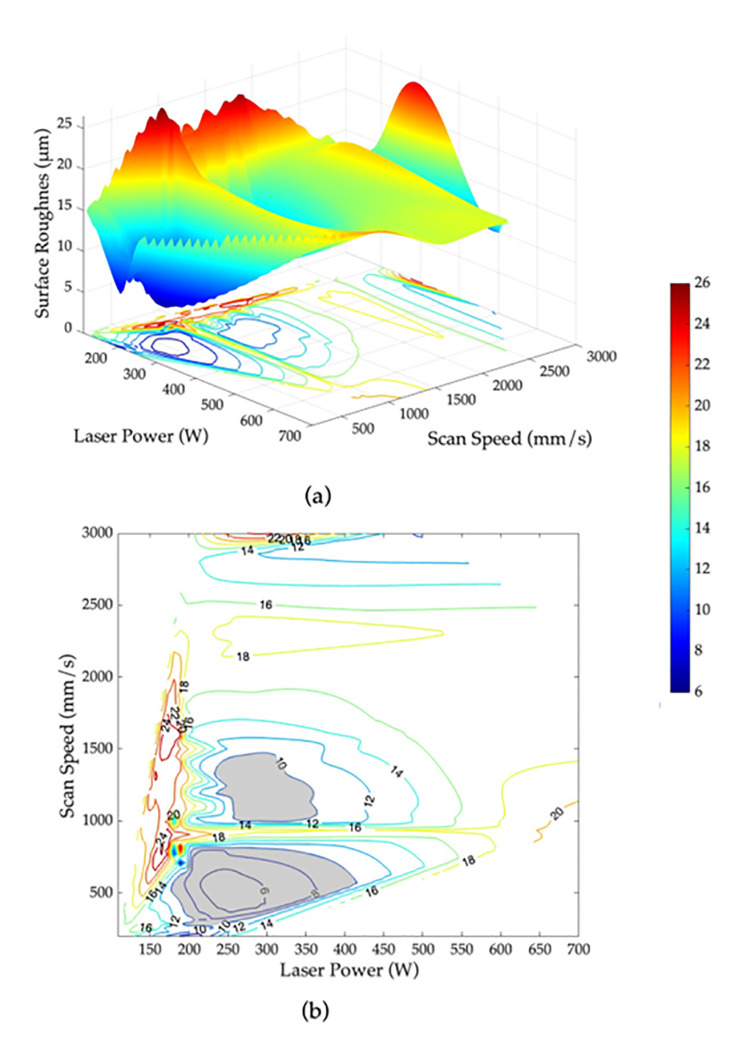
Surface roughness as a function of laser power and scan speed for AlSi10Mg parts using SLM (a) 3D contour map and (b) 2D contour map with the optimized processing window shown in grey for surface roughness <  10 µm.

**Fig 13:**
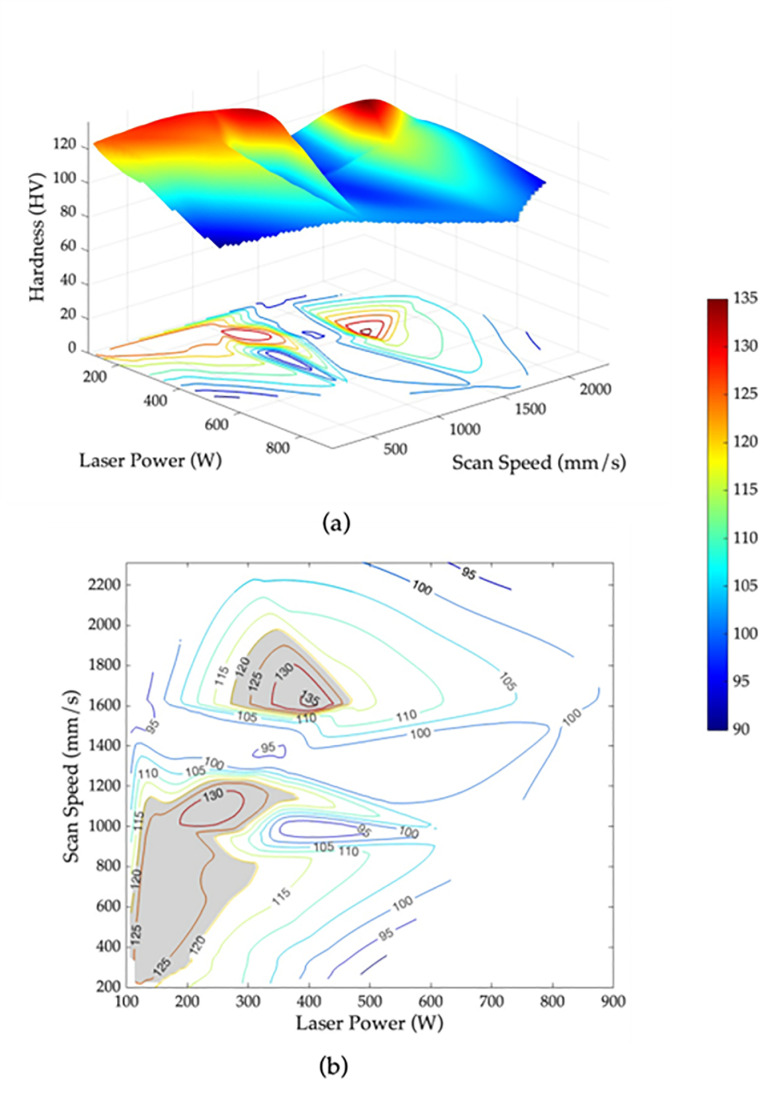
Hardness as a function of laser power and scan speed for AlSi10Mg parts using SLM (a) 3D contour map and (b) 2D contour map with the optimized processing window shown in grey for hardness >  120 HV.

Based on these findings, the importance of scan speed and laser power can be leveraged to optimize the properties of fabricated parts using SLM. The ANN models were employed to find the set of laser power and scan speed values that yield optimal results. The 3D contour maps of the relative density, surface roughness and hardness of the AlSi10Mg specimens are shown in Figs 11(a), 12(a), and 13(a), respectively. Based on the obtained 3D contour maps, the optimized processing window was defined for fabricating AlSi10Mg specimens with the following criteria: a relative density >  99%, surface roughness <  10 µm, and hardness >  120 HV. The optimized processing window is denoted by the grey areas in Figs 11(b), 12(b), and 13(b), respectively.

By merging the three individual optimized windows, as shown in Fig 13, an optimum region that overlaps all three criteria has been identified. This region indicates that the laser power between 250 to 300 W and scan speed range within 1000 to 1250 mm/s will provide an optimal relative density, surface roughness, and hardness for AlSi10Mg specimens fabricated using SLM. The results obtained are confirmed by the experimental data in Table 6. Incorporating this specific range of laser parameters during the manufacturing process ensures the production of high-quality AlSi10Mg parts with superior mechanical properties and performance.

**Fig 14:**
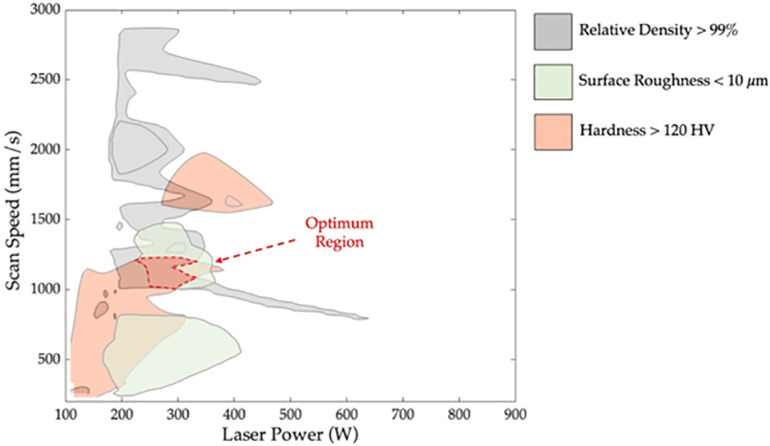
Optimum Region for AlSi10Mg parts using SLM with the merged criteria of relative density >  99%, surface roughness <  10 µm, and hardness >  120 HV.

## 4. Conclusions

This study examines the relative density, surface roughness and hardness of AlSi10Mg fabricated by SLM by combining literature data with experimental data. An analysis of the correlation between input process parameters, including laser power, scan speed, hatch distance, layer thickness, and the resulting relative density, surface roughness and hardness is studied. Five machine learning algorithms such as ANN, SVR, KRR, RF, and lasso regression were applied, and comprehensively analyzed and compared.

- Comparing the five ML algorithms for the relative density, surface roughness, and hardness, ANN and SVR outperformed the other algorithms for relative density and hardness. However, for surface roughness, ANN and RF outperformed the other models. ANN showed more predictability for the three properties of the AlSi10Mg fabricated parts as a function of the process parameters.
- Based and feature importance analysis using ANN, it was found that scan speed and laser power were the most significant features for relative density and hardness. Whereas for surface roughness, scan speed and layer thickness have a strong effect on the surface of the fabricated parts.
- Leveraging the significance of scan speed and laser power, an optimum region for laser power and scan speed has been identified. Specifically, a laser power between 250 to 300 W and a scan speed range within 1000 to 1250 mm/s will provide an optimal relative density near 100%, surface roughness smaller than 10 µm, and microhardness larger than 120 HV for AlSi10Mg specimens fabricated using SLM.
- This research provides valuable tools for additive manufacturing designers by predicting the relative density, surface roughness, and hardness of 3D-printed materials based on input process parameters. The machine learning models developed offer a cost-effective way to minimize experimental trials, prevent pore formation, improve surface quality, and enhance hardness through optimized parameter selection. Future research can leverage these predictive models with multi-objective optimization techniques to determine the optimal process parameters.

## Future work

Future research will extend these predictive models through multi-objective optimization techniques to balance surface roughness, hardness, and density outcomes. Due to the complexity of optimizing multiple material characteristics simultaneously, a dedicated study will address these intricate trade-offs and provide deeper insights into achieving optimal combinations of material properties for improved additive manufacturing performance.

## Supporting information

S1 File Supplementary data and figures. This file contains: **Tables S1-S3**: Summarized data from the literature for relative density, surface roughness, and hardness of AlSi10Mg fabricated parts using SLM. **Figures S1-S3**: Plots illustrating the relationships between process parameters (laser power, layer thickness, scan speed, hatch distance, and energy density) and material properties (relative density, surface roughness, and hardness). **Detailed Methodology**: Additional experimental setup, data preprocessing, and analysis protocols.
(ZIP)

S1 Checklist PLOSOne Human Subjects Research Checklist. (DOCX)
