# Supplementary material for: Prediction of porosity, hardness and surface roughness in additive manufactured AlSi10Mg samples
Source: PLoS One. 2025 Mar 10;20(3):e0316600. doi: 10.1371/journal.pone.0316600 (PMC11892887; doi:10.1371/journal.pone.0316600)
Supplement: S1 File — This file contains: Tables S1-S3: Summarized data from the literature for relative density, surface roughness, and hardness of AlSi10Mg fabricated parts using SLM. Figures S1-S3: Plots illustrating the relationships between process parameters (laser power, layer thickness, scan speed, hatch distance, and energy density) and material properties (relative density, surface roughness, and hardness). Detailed Methodology: Additional experimental setup, data preprocessing, and analysis protocols. (ZIP) [file pone.0316600.s001.zip › PLOSOne_Human_Subjects_Research_Checklist.docx]

**Human Participants Research Checklist**

***Complete the following if your study involved human participants or human participants’ data. These questions should be addressed for prospective and retrospective studies.***

1. Did you obtain ethics approval for this study?
   - If yes, please upload (file type “Other”) the original approval document you received from your ethics committee. If the original document is in another language, please also provide an English translation.

___ Uploaded X N/A (details provided below)

- - If you did not obtain ethical approval, please explain why this was not required below.

Ethical approval was not required for this study because:

1. The research is based solely on aggregated experimental and literature data related to material properties and manufacturing processes.
2. The study does not involve human participants, animals, or data derived from humans.
3. No identifiable personal data or sensitive participant-level information is included in the study or supporting materials.
4. The data used are publicly available or previously published in peer-reviewed articles, which are appropriately cited in our manuscript.

We confirm that our study fully complies with the ethical requirements of your journal, and no aspect of the research necessitated ethical review or approval.

1. If you prospectively recruited human participants for the study – for example, you conducted a clinical trial, distributed questionnaires, or obtained tissues, data or samples for the purposes of this study, please report in the Methods:
   1. the day, month and year of the **start and end** of the recruitment period for this study.
   2. whether participants provided informed consent, and if so, what type was obtained (for instance, written or verbal, and if verbal, how it was documented and witnessed). If your study included minors, state whether you obtained consent from parents or guardians. If the need for consent was waived by the ethics committee, please include this information.

___ Completed X N/A

1. If you are reporting a retrospective study of medical records or archived samples, please report in the Methods section:
2. the day, month and year when the data were accessed for research purposes
3. whether authors had access to information that could identify individual participants during or after data collection

___ Completed X N/A
