# Supplementary material for: Prediction of porosity, hardness and surface roughness in additive manufactured AlSi10Mg samples
Source: PLoS One. 2025 Mar 10;20(3):e0316600. doi: 10.1371/journal.pone.0316600 (PMC11892887; doi:10.1371/journal.pone.0316600)
Supplement: S1 File — This file contains: Tables S1-S3: Summarized data from the literature for relative density, surface roughness, and hardness of AlSi10Mg fabricated parts using SLM. Figures S1-S3: Plots illustrating the relationships between process parameters (laser power, layer thickness, scan speed, hatch distance, and energy density) and material properties (relative density, surface roughness, and hardness). Detailed Methodology: Additional experimental setup, data preprocessing, and analysis protocols. (ZIP) [file pone.0316600.s001.zip › S1_File.docx]

**Supplementary Document**

**1. Data from Literature**

The literature data used in this study were primarily collected from studies that focused on the relative density, surface roughness, and hardness of AlSi10Mg fabricated parts using SLM with varying process parameters. These parameters include laser power, scan speed, hatch distance, and layer thickness. The data collected from the literature for relative density, surface roughness, and hardness are included in Tables S1, S2, and S3, respectively.

| **Table S1.** Data from literature for relative density. | | | | | |
| --- | --- | --- | --- | --- | --- |
| **Author** | **Laser Power (W)** | **Scan Speed (mm/s)** | **Hatch Distance (mm)** | **Layer Thickness (mm)** | **Relative Density (%)** |
|  |  |  |  |  |  |
| Maamoun et al. [1] | 370 | 1000 | 0.19 | 0.03 | 99.3 |
|  | 370 | 1300 | 0.15 | 0.03 | 99.5 |
|  | 370 | 1300 | 0.19 | 0.03 | 99.9 |
|  | 350 | 1300 | 0.19 | 0.03 | 99.9 |
|  | 370 | 1500 | 0.19 | 0.03 | 99.6 |
|  | 300 | 1300 | 0.19 | 0.03 | 99.3 |
|  | 370 | 1300 | 0.25 | 0.03 | 99.0 |
|  | 200 | 1300 | 0.19 | 0.03 | 96.9 |
| Han et al.  [2] | 200 | 600 | 0.04 | 0.02 | 94.6 |
|  | 200 | 800 | 0.04 | 0.02 | 97.7 |
|  | 200 | 1000 | 0.04 | 0.02 | 98.8 |
|  | 200 | 1200 | 0.04 | 0.02 | 99.1 |
|  | 200 | 1400 | 0.04 | 0.02 | 99.8 |
|  | 200 | 1600 | 0.04 | 0.02 | 99.6 |
|  | 200 | 1800 | 0.04 | 0.02 | 99.1 |
|  | 200 | 2000 | 0.04 | 0.02 | 99.5 |
|  | 200 | 2200 | 0.04 | 0.02 | 99.3 |
|  | 200 | 2400 | 0.04 | 0.02 | 99.3 |
|  | 200 | 2600 | 0.04 | 0.02 | 99.4 |
|  | 200 | 2800 | 0.04 | 0.02 | 99.2 |
|  | 200 | 3000 | 0.04 | 0.02 | 99.2 |
|  | 200 | 600 | 0.06 | 0.02 | 97.8 |
|  | 200 | 800 | 0.06 | 0.02 | 98.6 |
|  | 200 | 1000 | 0.06 | 0.02 | 99.6 |
|  | 200 | 1200 | 0.06 | 0.02 | 99.4 |
|  | 200 | 1400 | 0.06 | 0.02 | 99.5 |
|  | 200 | 1600 | 0.06 | 0.02 | 99.6 |
|  | 200 | 1800 | 0.06 | 0.02 | 99.7 |
|  | 200 | 2000 | 0.06 | 0.02 | 99.5 |
|  | 200 | 2200 | 0.06 | 0.02 | 99.5 |
|  | 200 | 2400 | 0.06 | 0.02 | 99.7 |
|  | 200 | 2600 | 0.06 | 0.02 | 99.7 |
|  | 200 | 2800 | 0.06 | 0.02 | 99.5 |
|  | 200 | 3000 | 0.06 | 0.02 | 99.5 |
|  | 200 | 600 | 0.08 | 0.02 | 99.0 |
|  | 200 | 800 | 0.08 | 0.02 | 98.7 |
|  | 200 | 1000 | 0.08 | 0.02 | 99.0 |
|  | 200 | 1200 | 0.08 | 0.02 | 99.4 |
|  | 200 | 1400 | 0.08 | 0.02 | 99.5 |
|  | 200 | 1600 | 0.08 | 0.02 | 99.5 |
|  | 200 | 1800 | 0.08 | 0.02 | 99.6 |
|  | 200 | 2000 | 0.08 | 0.02 | 99.5 |
|  | 200 | 2200 | 0.08 | 0.02 | 99.5 |
|  | 200 | 2400 | 0.08 | 0.02 | 99.6 |
|  | 200 | 2600 | 0.08 | 0.02 | 99.5 |
|  | 200 | 2800 | 0.08 | 0.02 | 99.6 |
|  | 200 | 3000 | 0.08 | 0.02 | 99.6 |
|  | 200 | 600 | 0.1 | 0.02 | 98.4 |
|  | 200 | 800 | 0.1 | 0.02 | 98.7 |
|  | 200 | 1000 | 0.1 | 0.02 | 99.6 |
|  | 200 | 1200 | 0.1 | 0.02 | 99.5 |
|  | 200 | 1400 | 0.1 | 0.02 | 99.3 |
|  | 200 | 1600 | 0.1 | 0.02 | 99.6 |
|  | 200 | 1800 | 0.1 | 0.02 | 99.7 |
|  | 200 | 2000 | 0.1 | 0.02 | 99.2 |
|  | 200 | 2200 | 0.1 | 0.02 | 99.3 |
|  | 200 | 2400 | 0.1 | 0.02 | 99.3 |
|  | 200 | 2600 | 0.1 | 0.02 | 99.5 |
|  | 200 | 2800 | 0.1 | 0.02 | 99.7 |
|  | 200 | 3000 | 0.1 | 0.02 | 99.7 |
| Pei et al.  [3] | 180 | 600 | 0.05 | 0.04 | 94.1 |
|  | 180 | 800 | 0.05 | 0.04 | 97.8 |
|  | 180 | 1000 | 0.05 | 0.04 | 99.6 |
|  | 180 | 1400 | 0.05 | 0.04 | 91.8 |
|  | 180 | 1000 | 0.07 | 0.04 | 94.8 |
|  | 180 | 1000 | 0.06 | 0.04 | 96.7 |
|  | 150 | 600 | 0.05 | 0.04 | 95.6 |
|  | 150 | 800 | 0.05 | 0.04 | 99.0 |
|  | 150 | 1000 | 0.05 | 0.04 | 97.0 |
|  | 150 | 1400 | 0.05 | 0.04 | 89.7 |
| Sing et al.  [4] | 350 | 1140 | 0.17 | 0.05 | 98.1 |
| Read et al.  [5] | 125 | 1675 | 0.0525 | 0.05 | 83.9 |
|  | 125 | 1675 | 0.0975 | 0.05 | 75.3 |
|  | 125 | 1025 | 0.0975 | 0.05 | 90.6 |
|  | 150 | 1350 | 0.12 | 0.05 | 89.2 |
|  | 125 | 1675 | 0.0975 | 0.05 | 70.1 |
|  | 150 | 700 | 0.075 | 0.05 | 89.6 |
|  | 150 | 1350 | 0.075 | 0.05 | 90.1 |
|  | 125 | 1675 | 0.0525 | 0.05 | 84.6 |
|  | 175 | 1025 | 0.0975 | 0.05 | 98.3 |
|  | 175 | 1675 | 0.0975 | 0.05 | 94.5 |
|  | 125 | 1025 | 0.0525 | 0.05 | 88.2 |
|  | 150 | 1350 | 0.03 | 0.05 | 89.5 |
|  | 125 | 1025 | 0.0525 | 0.05 | 85.9 |
|  | 150 | 1350 | 0.075 | 0.05 | 92.5 |
|  | 100 | 1350 | 0.075 | 0.05 | 79.5 |
|  | 150 | 1350 | 0.075 | 0.05 | 89.9 |
|  | 175 | 1025 | 0.0525 | 0.05 | 96.5 |
|  | 125 | 1025 | 0.0975 | 0.05 | 90.7 |
|  | 175 | 1675 | 0.0525 | 0.05 | 93.6 |
|  | 175 | 1675 | 0.0975 | 0.05 | 86.9 |
|  | 200 | 1350 | 0.075 | 0.05 | 99.2 |
|  | 150 | 2000 | 0.075 | 0.05 | 82.0 |
|  | 175 | 1675 | 0.0525 | 0.05 | 93.2 |
|  | 150 | 1350 | 0.075 | 0.05 | 94.5 |
|  | 150 | 1350 | 0.075 | 0.05 | 92.7 |
|  | 175 | 1025 | 0.0975 | 0.05 | 99.2 |
|  | 175 | 1025 | 0.0525 | 0.05 | 97.6 |
| Kempen et al.  [6] | 200 | 1400 | 0.075 | 0.03 | 98.9 |
|  | 200 | 1400 | 0.09 | 0.03 | 99.0 |
|  | 200 | 1400 | 0.105 | 0.03 | 99.0 |
|  | 200 | 1400 | 0.12 | 0.03 | 99.0 |
|  | 200 | 1400 | 0.135 | 0.03 | 98.9 |
|  | 170 | 800 | 0.105 | 0.03 | 99.1 |
|  | 170 | 1000 | 0.105 | 0.03 | 99.3 |
|  | 170 | 1200 | 0.105 | 0.03 | 99.1 |
|  | 170 | 1400 | 0.105 | 0.03 | 98.5 |
|  | 170 | 1600 | 0.105 | 0.03 | 97.7 |
|  | 180 | 900 | 0.105 | 0.03 | 99.0 |
|  | 180 | 1200 | 0.105 | 0.03 | 99.2 |
|  | 180 | 1300 | 0.105 | 0.03 | 99.2 |
|  | 180 | 1400 | 0.105 | 0.03 | 98.9 |
|  | 180 | 1500 | 0.105 | 0.03 | 98.7 |
|  | 190 | 800 | 0.105 | 0.03 | 98.3 |
|  | 190 | 1000 | 0.105 | 0.03 | 98.8 |
|  | 190 | 1200 | 0.105 | 0.03 | 99.2 |
|  | 190 | 1400 | 0.105 | 0.03 | 99.2 |
|  | 190 | 1600 | 0.105 | 0.03 | 98.9 |
|  | 200 | 200 | 0.105 | 0.03 | 94.7 |
|  | 200 | 400 | 0.105 | 0.03 | 96.8 |
|  | 200 | 500 | 0.105 | 0.03 | 97.6 |
|  | 200 | 600 | 0.105 | 0.03 | 98.3 |
|  | 200 | 700 | 0.105 | 0.03 | 98.3 |
|  | 200 | 800 | 0.105 | 0.03 | 98.8 |
|  | 200 | 900 | 0.105 | 0.03 | 98.8 |
|  | 200 | 1000 | 0.105 | 0.03 | 99.1 |
|  | 200 | 1100 | 0.105 | 0.03 | 98.9 |
|  | 200 | 1200 | 0.105 | 0.03 | 99.1 |
|  | 200 | 1300 | 0.105 | 0.03 | 99.2 |
|  | 200 | 1400 | 0.105 | 0.03 | 99.3 |
|  | 200 | 1500 | 0.105 | 0.03 | 99.2 |
| Aboulkhair et al.  [7] | 100 | 500 | 0.05 | 0.04 | 97.8 |
|  | 100 | 500 | 0.1 | 0.04 | 95.8 |
|  | 100 | 500 | 0.15 | 0.04 | 94.5 |
|  | 100 | 500 | 0.2 | 0.04 | 89 |
|  | 100 | 500 | 0.25 | 0.04 | 76.9 |
|  | 100 | 250 | 0.05 | 0.04 | 95.6 |
|  | 100 | 500 | 0.05 | 0.04 | 97.7 |
|  | 100 | 750 | 0.05 | 0.04 | 96.8 |
|  | 100 | 1000 | 0.05 | 0.04 | 96.9 |
|  | 100 | 250 | 0.1 | 0.04 | 96.3 |
| Yap et al.  [8] | 350 | 1140 | 0.17 | 0.05 | 99.8 |
| Raus et al.  [9] | 350 | 1650 | 0.13 | 0.03 | 99.0 |
|  | 350 | 1650 | 0.13 | 0.03 | 99.1 |
|  | 350 | 1650 | 0.13 | 0.03 | 99.1 |
|  | 350 | 1650 | 0.13 | 0.03 | 99.1 |
|  | 350 | 1650 | 0.13 | 0.03 | 99.0 |
|  | 350 | 1650 | 0.13 | 0.03 | 99.0 |
|  | 350 | 1650 | 0.13 | 0.03 | 99.1 |
|  | 350 | 1650 | 0.13 | 0.03 | 99.0 |
| Kan. et al.  [10] | 350 | 930 | 0.17 | 0.05 | 96.4 |
|  | 350 | 930 | 0.17 | 0.05 | 96.1 |
|  | 350 | 930 | 0.17 | 0.05 | 96.5 |
|  | 350 | 930 | 0.17 | 0.05 | 96.2 |
| Bai et al.  [11] | 625 | 1400 | 0.35 | 0.06 | 93.8 |
|  | 950 | 1400 | 0.35 | 0.06 | 99.2 |
|  | 788 | 2300 | 0.35 | 0.06 | 87.0 |
|  | 463 | 2300 | 0.35 | 0.06 | 72.0 |
|  | 300 | 1400 | 0.35 | 0.06 | 72.1 |
|  | 463 | 500 | 0.35 | 0.06 | 99.6 |
|  | 788 | 500 | 0.35 | 0.06 | 94.5 |
|  | 788 | 1700 | 0.4 | 0.06 | 90.7 |
|  | 463 | 1700 | 0.4 | 0.06 | 75.7 |
|  | 625 | 800 | 0.4 | 0.06 | 99.2 |
|  | 788 | 1099 | 0.3 | 0.06 | 99.8 |
|  | 463 | 1099 | 0.3 | 0.06 | 92.9 |
|  | 625 | 2000 | 0.3 | 0.06 | 85.6 |
|  | 300 | 800 | 0.3 | 0.06 | 88.4 |
| Wang et al.  [12] | 400 | 250 | 0.13 | 0.025 | 94.3 |
|  | 400 | 270 | 0.13 | 0.025 | 96.7 |
|  | 400 | 290 | 0.13 | 0.025 | 96.5 |
|  | 400 | 310 | 0.13 | 0.025 | 96.6 |
|  | 400 | 330 | 0.13 | 0.025 | 96.2 |
|  | 400 | 250 | 0.13 | 0.025 | 95.7 |
|  | 400 | 270 | 0.13 | 0.025 | 96.1 |
|  | 400 | 290 | 0.13 | 0.025 | 96.6 |
|  | 400 | 310 | 0.13 | 0.025 | 97.2 |
|  | 400 | 330 | 0.13 | 0.025 | 96.4 |
|  | 400 | 250 | 0.13 | 0.025 | 95.8 |
|  | 400 | 270 | 0.13 | 0.025 | 96.6 |
|  | 400 | 290 | 0.13 | 0.025 | 96.7 |
|  | 400 | 310 | 0.13 | 0.025 | 96.7 |
|  | 400 | 330 | 0.13 | 0.025 | 96.5 |
|  | 400 | 250 | 0.13 | 0.025 | 96.0 |
|  | 400 | 270 | 0.13 | 0.025 | 96.5 |
|  | 400 | 290 | 0.13 | 0.025 | 97.7 |
|  | 400 | 310 | 0.13 | 0.025 | 97.6 |
|  | 400 | 330 | 0.13 | 0.025 | 96.9 |
|  | 400 | 250 | 0.13 | 0.025 | 95.9 |
|  | 400 | 270 | 0.13 | 0.025 | 97.4 |
|  | 400 | 290 | 0.13 | 0.025 | 97.8 |
|  | 400 | 310 | 0.13 | 0.025 | 97.8 |
|  | 400 | 330 | 0.13 | 0.025 | 97.0 |

| **Table S2.** Data from literature for surface roughness. | | | | | |
| --- | --- | --- | --- | --- | --- |
| **Author** | **Laser Power (W)** | **Scan Speed (mm/s)** | **Hatch Distance (mm)** | **Layer Thickness (mm)** | **Surface Roughness**  **(μm)** |
|  |  |  |  |  |  |
| Han et al.  [2] | 200 | 600 | 0.04 | 0.02 | 7.4 |
|  | 200 | 800 | 0.04 | 0.02 | 6.5 |
|  | 200 | 1000 | 0.04 | 0.02 | 11.8 |
|  | 200 | 1200 | 0.04 | 0.02 | 16.0 |
|  | 200 | 1400 | 0.04 | 0.02 | 10.4 |
|  | 200 | 1600 | 0.04 | 0.02 | 10.6 |
|  | 200 | 1800 | 0.04 | 0.02 | 14.6 |
|  | 200 | 2000 | 0.04 | 0.02 | 10.7 |
|  | 200 | 2200 | 0.04 | 0.02 | 8.6 |
|  | 200 | 2400 | 0.04 | 0.02 | 18.4 |
|  | 200 | 2600 | 0.04 | 0.02 | 12.4 |
|  | 200 | 2800 | 0.04 | 0.02 | 12.5 |
|  | 200 | 3000 | 0.04 | 0.02 | 12.2 |
|  | 200 | 600 | 0.06 | 0.02 | 6.4 |
|  | 200 | 800 | 0.06 | 0.02 | 6.6 |
|  | 200 | 1000 | 0.06 | 0.02 | 13.5 |
|  | 200 | 1200 | 0.06 | 0.02 | 13.8 |
|  | 200 | 1400 | 0.06 | 0.02 | 12.8 |
|  | 200 | 1600 | 0.06 | 0.02 | 15.0 |
|  | 200 | 1800 | 0.06 | 0.02 | 18.8 |
|  | 200 | 2000 | 0.06 | 0.02 | 19.0 |
|  | 200 | 2200 | 0.06 | 0.02 | 17.8 |
|  | 200 | 2400 | 0.06 | 0.02 | 14.2 |
|  | 200 | 2600 | 0.06 | 0.02 | 17.3 |
|  | 200 | 2800 | 0.06 | 0.02 | 15.4 |
|  | 200 | 3000 | 0.06 | 0.02 | 10.9 |
|  | 200 | 600 | 0.08 | 0.02 | 5.4 |
|  | 200 | 800 | 0.08 | 0.02 | 14.3 |
|  | 200 | 1000 | 0.08 | 0.02 | 15.4 |
|  | 200 | 1200 | 0.08 | 0.02 | 9.3 |
|  | 200 | 1400 | 0.08 | 0.02 | 13.2 |
|  | 200 | 1600 | 0.08 | 0.02 | 14.4 |
|  | 200 | 1800 | 0.08 | 0.02 | 11.7 |
|  | 200 | 2000 | 0.08 | 0.02 | 10.5 |
|  | 200 | 2200 | 0.08 | 0.02 | 14.3 |
|  | 200 | 2400 | 0.08 | 0.02 | 19.8 |
|  | 200 | 2600 | 0.08 | 0.02 | 17.3 |
|  | 200 | 2800 | 0.08 | 0.02 | 16.5 |
|  | 200 | 3000 | 0.08 | 0.02 | 22.8 |
|  | 200 | 600 | 0.1 | 0.02 | 8.4 |
|  | 200 | 800 | 0.1 | 0.02 | 8.8 |
|  | 200 | 1000 | 0.1 | 0.02 | 8.9 |
|  | 200 | 1200 | 0.1 | 0.02 | 7.3 |
|  | 200 | 1400 | 0.1 | 0.02 | 6.2 |
|  | 200 | 1600 | 0.1 | 0.02 | 4.9 |
|  | 200 | 1800 | 0.1 | 0.02 | 13.2 |
|  | 200 | 2000 | 0.1 | 0.02 | 11.2 |
|  | 200 | 2200 | 0.1 | 0.02 | 20.9 |
|  | 200 | 2400 | 0.1 | 0.02 | 16.7 |
|  | 200 | 2600 | 0.1 | 0.02 | 7.4 |
|  | 200 | 2800 | 0.1 | 0.02 | 9.0 |
|  | 200 | 3000 | 0.1 | 0.02 | 6.3 |
| Pei et al.  [3] | 180 | 800 | 0.05 | 0.04 | 14.2 |
|  | 180 | 1000 | 0.05 | 0.04 | 11.1 |
|  | 180 | 1400 | 0.05 | 0.04 | 20.2 |
| Kempen et al.  [6] | 170 | 800 | 0.105 | 0.03 | 23.9 |
|  | 170 | 1000 | 0.105 | 0.03 | 24.0 |
|  | 170 | 1200 | 0.105 | 0.03 | 23.2 |
|  | 170 | 1400 | 0.105 | 0.03 | 21.9 |
|  | 170 | 1600 | 0.105 | 0.03 | 23.9 |
|  | 180 | 900 | 0.105 | 0.03 | 21.6 |
|  | 180 | 1100 | 0.105 | 0.03 | 21.4 |
|  | 180 | 1300 | 0.105 | 0.03 | 20.8 |
|  | 180 | 1400 | 0.105 | 0.03 | 20.9 |
|  | 180 | 1500 | 0.105 | 0.03 | 26.1 |
|  | 190 | 800 | 0.105 | 0.03 | 26.2 |
|  | 190 | 1000 | 0.105 | 0.03 | 19.4 |
|  | 190 | 1200 | 0.105 | 0.03 | 17.8 |
|  | 190 | 1400 | 0.105 | 0.03 | 21.1 |
|  | 190 | 1600 | 0.105 | 0.03 | 23.0 |
|  | 200 | 1100 | 0.105 | 0.03 | 16.4 |
|  | 200 | 1200 | 0.105 | 0.03 | 17.7 |
|  | 200 | 1300 | 0.105 | 0.03 | 18.2 |
|  | 200 | 1400 | 0.105 | 0.03 | 20.9 |
| Poncelet et al.  [13] | 274 | 200 | 0.1 | 0.03 | 14.3 |
|  | 246 | 200 | 0.1 | 0.03 | 12.8 |
|  | 219 | 200 | 0.1 | 0.03 | 7.3 |
|  | 205 | 200 | 0.1 | 0.03 | 5.9 |
|  | 164 | 200 | 0.1 | 0.03 | 8.6 |
|  | 137 | 200 | 0.1 | 0.03 | 12.5 |
|  | 109 | 200 | 0.1 | 0.03 | 13.2 |
|  | 273 | 200 | 0.1 | 0.03 | 14.3 |
|  | 273 | 300 | 0.1 | 0.03 | 9.9 |
|  | 273 | 400 | 0.1 | 0.03 | 4.5 |
|  | 273 | 500 | 0.1 | 0.03 | 4.8 |
|  | 273 | 800 | 0.1 | 0.03 | 8.4 |
|  | 273 | 1000 | 0.1 | 0.03 | 9.0 |
|  | 273 | 1200 | 0.1 | 0.03 | 7.5 |
|  | 273 | 1500 | 0.1 | 0.03 | 7.0 |

| **Table S3.** Data from literature for hardness. | | | | | |
| --- | --- | --- | --- | --- | --- |
| **Author** | **Laser Power (W)** | **Scan Speed (mm/s)** | **Hatch Distance (mm)** | **Layer Thickness (mm)** | **Hardness**  **(HV)** |
|  |  |  |  |  |  |
| Maamoun et al. [1] | 370 | 1000 | 0.19 | 0.03 | 116 |
|  | 370 | 1300 | 0.15 | 0.03 | 117 |
|  | 370 | 1300 | 0.19 | 0.03 | 115.6 |
|  | 350 | 1300 | 0.19 | 0.03 | 115.4 |
|  | 370 | 1500 | 0.19 | 0.03 | 114.6 |
|  | 300 | 1300 | 0.19 | 0.03 | 115.2 |
|  | 370 | 1300 | 0.25 | 0.03 | 118.4 |
|  | 200 | 1300 | 0.19 | 0.03 | 118.6 |
| Poncelet et al.  [13] | 274 | 200 | 0.1 | 0.03 | 108.4 |
|  | 246 | 200 | 0.1 | 0.03 | 110.2 |
|  | 219 | 200 | 0.1 | 0.03 | 112.0 |
|  | 205 | 200 | 0.1 | 0.03 | 115.0 |
|  | 164 | 200 | 0.1 | 0.03 | 116.2 |
|  | 137 | 200 | 0.1 | 0.03 | 119.3 |
|  | 109 | 200 | 0.1 | 0.03 | 127.9 |
|  | 273 | 200 | 0.1 | 0.03 | 108.5 |
|  | 273 | 300 | 0.1 | 0.03 | 114.2 |
|  | 273 | 400 | 0.1 | 0.03 | 115.1 |
|  | 273 | 500 | 0.1 | 0.03 | 120.2 |
|  | 273 | 800 | 0.1 | 0.03 | 128.8 |
|  | 273 | 1000 | 0.1 | 0.03 | 132.2 |
|  | 273 | 1200 | 0.1 | 0.03 | 125.7 |
|  | 273 | 1500 | 0.1 | 0.03 | 128.3 |
| Yusuf et al.  [14] | 400 | 1600 | 0.2 | 0.02 | 145 |
|  | 400 | 1600 | 0.2 | 0.02 | 136 |
| Tridello et al.  [15] | 350 | 1150 | 0.17 | 0.05 | 132 |
|  | 350 | 1150 | 0.17 | 0.05 | 146 |
| Mfusi et al.  [16] | 150 | 1000 | 0.05 | 0.05 | 128 |
|  | 150 | 1000 | 0.05 | 0.05 | 127 |
|  | 150 | 1000 | 0.05 | 0.05 | 126 |

**2. Analysis of Data Plots**

The plots in Figures S1, S2, and S3 show how the relative density, surface roughness, and hardness, respectively, change as the laser power, layer thickness, scan speed, hatch distance, and energy density that combines all the four process parameters vary. The plots show that the properties vary depending on the process parameters used. These plots are based on a combination of literature data and the experimental results of this study. The compiled dataset for relative density, surface roughness, and hardness includes laser power between 100 and 950 W, layer thickness ranging between 0.02 and 0.06 mm, scanning speed between 200 and 3000 mm/s, and hatch distance between 0.03 and 0.4 mm. The relative density, surface roughness, and hardness of the samples tested ranges from 70% to 100%, 4 μm to 29 μm, and 65 HV to 146 HV, respectively.


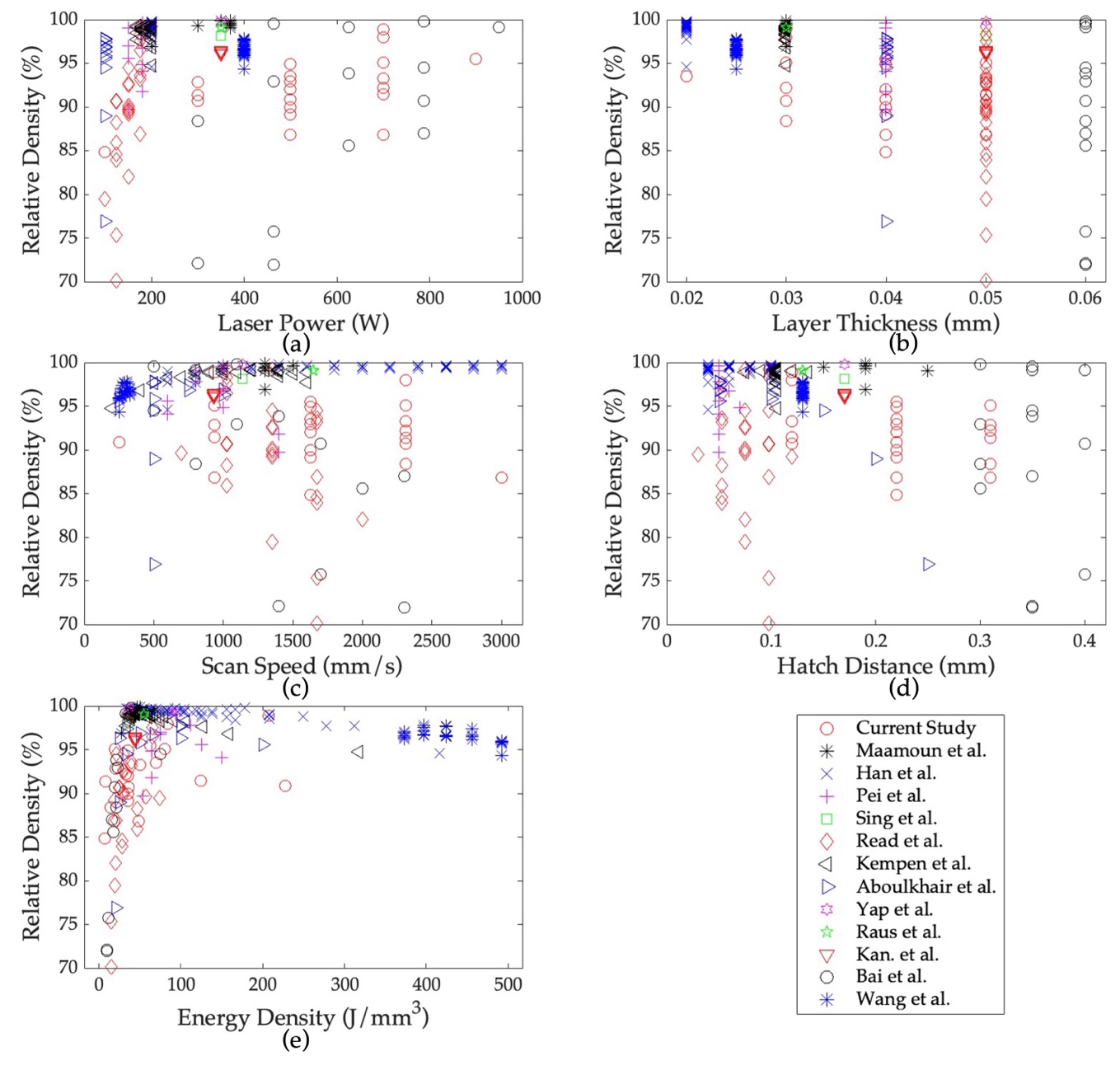


**Figure S1:** Plots of the relative density in relation to (**a**) laser power, (**b**) layer thickness, (**c**) scan speed, (**d**) hatch distance, and (**e**) energy density.

For relative density, Figure S1(a) shows that the relative density exceeds 85% as the laser power exceeds 600 W. More material is heated as laser power and thermal energy increase, above its melting point [17]. A higher laser power results in a sufficient melting of the substrate, thus increasing the relative density of part [18]. With respect to the layer thickness, Figure S1(b) shows that a high relative density is obtained when the layer thickness is less than 0.03 mm. The lower the layer thickness the more laser energy is concentrated on a smaller volume of material, resulting in sufficient powder melting. In contrast, if the powder layer is too thick, it would be difficult to create a melt pool with sufficient depth to melt and fuse the layers. [19]. Thus, as the layer thickness increases, the relative density decreases. This observation agrees with the study in [20]. Moreover, as shown in Figure S1(c), the relative density is high when the scan speed is lower than 1000 mm/s. At a very high scan speed, the laser might not be able to melt the powder enough, resulting in scan tracks with large pores that are not fully melted. This can cause a decrease in the density of the measured part. In Figure S1(d), The experimental results of various hatch distances and the measured relative densities are presented. There is no clear correlation between hatch distance and relative density. However, major points with high relative density are observed at a smaller hatch distance. Decreasing the hatch distance allows the laser to overlap sufficiently, resulting in a sufficient powder melting [19]. Lastly, Figure S1(e) shows that as the energy density of the fabricated part increases the relative density also increases.

The surface roughness of the printed parts is influenced by various process parameters as shown in the plots in Figure S2. Specifically, in Figure S2(a) there is no clear correlation between the surface roughness and laser power. However, insufficient laser power will cause the powder to not melt and spread completely. This results in an uneven melting channel that may not be continuous or smooth, causing the surface of the material to become wavy and rough [20]. On the other hand, using a very high laser power can cause excessive melting of the powder particles can occur, in which it results in a low surface quality. In Figure S2(b), it is observed that lower surface roughness is achieved using smaller layer thickness. Thicker layers will not be melted as effectively as thinner layers, resulting in a rougher surface finish. Furthermore, Figure S2(c) also shows no clear correlation between the scan speed and the surface roughness. But higher surface roughness is observed at scan speeds between 1000 mm/s to 1500 mm/s. For a given laser power, surface roughness can reach a minimum at a certain scan speed [6], too high or too low scan speed can result in rougher surface. However, the optimal scan speed for achieving the desired surface quality can vary based on other parameters of the process and the material being used. Based on Figure S2(d), a low surface roughness is observed when the hatch distance is lower than 0.1 mm. Larger hatch distances will result to insufficient powder melting, that can result in incomplete fusion and weak bonding, increasing the surface roughness. In Figure S2(e), there was no clear correlation found between the energy density and surface roughness.

For the microhardness of AlSi10mg fabricated parts, it is observed in Figure S3(a) that the reduction in laser power results in a higher microhardness. This trend agrees with the study [1]. In Figure S3(b), the majority of the experiments used a constant layer thickness of 0.03 mm, resulting in no clear correlation between the layer thickness and the microhardness. However, studies [20], [21] found that as the layer thickness increased the microhardness decreased. The scan speed plot in Figure S3(c) shows that increasing the speed decreases the microhardness. When the scan speed is increased, the scan tracks may not be fully melted, resulting in the formation of large pores which will decrease the hardness [19]. In Figure S3(d) it is shown that increasing the hatch distance results in lower microhardness. This relationship was observed in several studies, where the increase in hatch distance results in higher porosity [22], and lower density [23], [24], resulting in a lower microhardness. Lastly, in Figure S3(e) it is shown that as the energy density decreases the hardness of the parts increases.

To summarize, a high laser power can result in less pores formation and decrease the hardness of the part. Moreover, decreasing the layer thickness would improve the relative density and result in a lower surface roughness. However, scan speed is observed to increase the relative density if maintained between 1000 mm/s and 1500 mm/s and increase the hardness at a higher speed. Lastly a lower hatch distance would result in a lower surface roughness and harder parts.


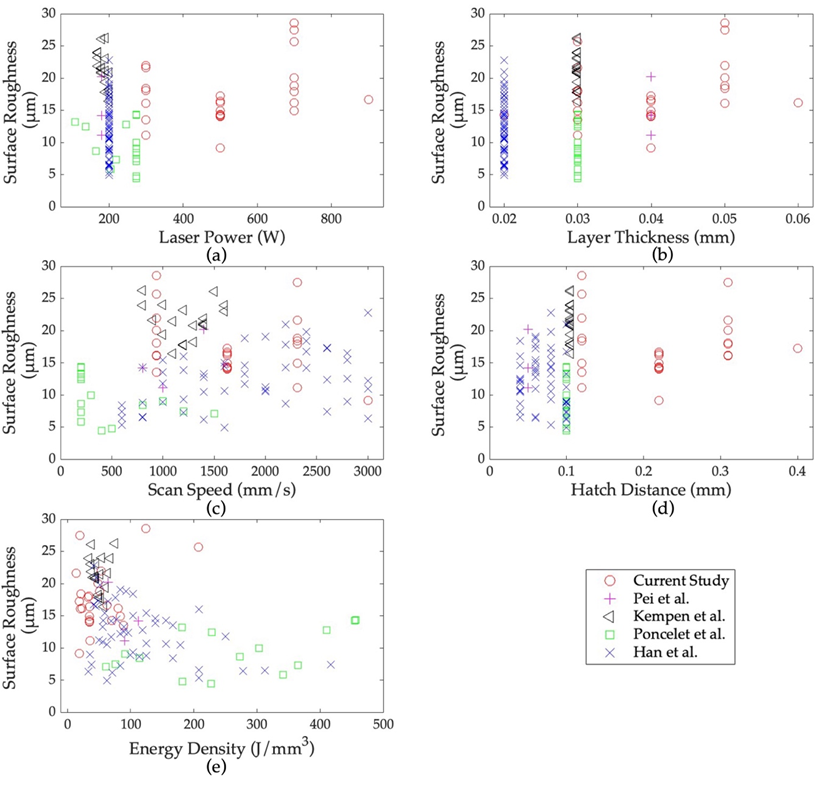


**Figure S2:** Plots of the surface roughness in relation to (**a**) laser power, (**b**) layer thickness, (**c**) scan speed, (**d**) hatch distance, and (**e**) energy density.


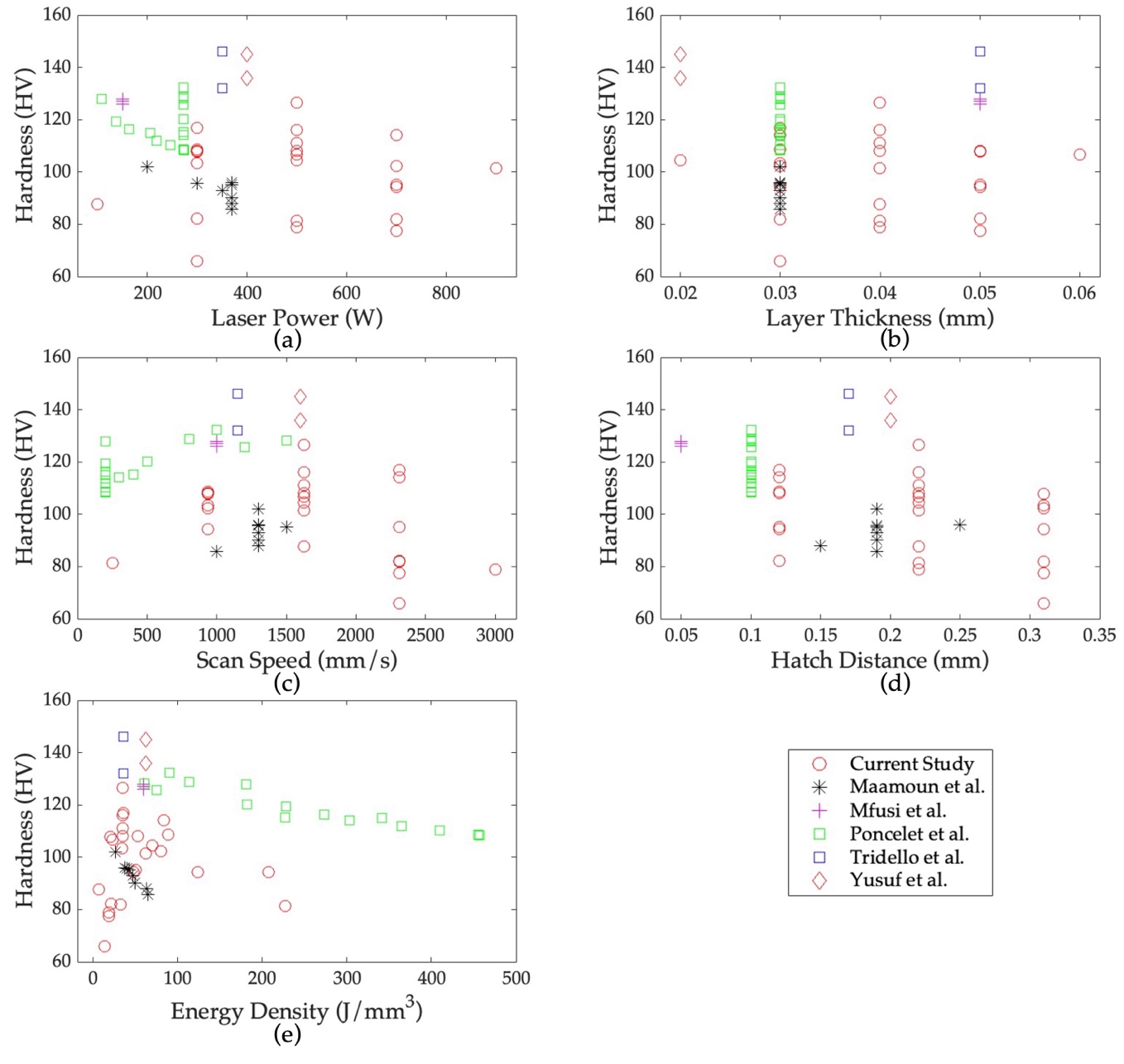


**Figure S3:** Plots of the hardness in relation to (**a**) laser power, (**b**) layer thickness, (**c**) scan speed, (**d**) hatch distance, and (**e**) energy density.

**References**

[1] A. Maamoun, Y. Xue, M. Elbestawi, and S. Veldhuis, “The Effect of Selective Laser Melting Process Parameters on the Microstructure and Mechanical Properties of Al6061 and AlSi10Mg Alloys,” *Materials*, vol. 12, no. 1, p. 12, 2018, doi: 10.3390/ma12010012.

[2] X. Han, H. Zhu, X. Nie, G. Wang, and X. Zeng, “Investigation on selective laser melting AlSi10Mg cellular lattice strut: Molten pool morphology, surface roughness and dimensional accuracy,” *Materials*, vol. 11, no. 3, 2018, doi: 10.3390/ma11030392.

[3] P. Wei *et al.*, “The AlSi10Mg samples produced by selective laser melting: single track, densification, microstructure and mechanical behavior,” *Appl Surf Sci*, vol. 408, pp. 38–50, 2017, doi: 10.1016/j.apsusc.2017.02.215.

[4] S. L. Sing, L. P. Lam, D. Q. Zhang, Z. H. Liu, and C. K. Chua, “Interfacial characterization of SLM parts in multi-material processing: Intermetallic phase formation between AlSi10Mg and C18400 copper alloy,” *Mater Charact*, vol. 107, pp. 220–227, 2015, doi: 10.1016/j.matchar.2015.07.007.

[5] N. Read, W. Wang, K. Essa, and M. M. Attallah, “Selective laser melting of AlSi10Mg alloy: Process optimisation and mechanical properties development,” *Mater Des*, vol. 65, pp. 417–424, 2015, doi: 10.1016/j.matdes.2014.09.044.

[6] K. Kempen, L. Thijs, E. Yasa, M. Badrossamay, W. Verheecke°, and J.-P. Kruth, “PROCESS OPTIMIZATION AND MICROSTRUCTURAL ANALYSIS FOR SELECTIVE LASER MELTING OF AlSi10Mg,” in *22nd Annual International Solid Freeform Fabrication Symposium - An Additive Manufacturing Conference*, 2011.

[7] N. T. Aboulkhair, N. M. Everitt, I. Ashcroft, and C. Tuck, “Reducing porosity in AlSi10Mg parts processed by selective laser melting,” *Addit Manuf*, vol. 1, pp. 77–86, 2014, doi: 10.1016/j.addma.2014.08.001.

[8] C. Y. Yap, C. K. Chua, and Z. L. Dong, “An effective analytical model of selective laser melting,” *Virtual Phys Prototyp*, vol. 11, no. 1, pp. 21–26, 2016, doi: 10.1080/17452759.2015.1133217.

[9] A. A. Raus, M. S. Wahab, M. Ibrahim, K. Kamarudin, A. Ahmed, and S. Shamsudin, “Mechanical and physical properties of AlSi10Mg processed through selective laser melting,” in *AIP Conference Proceedings*, American Institute of Physics Inc., 2017. doi: 10.1063/1.4981168.

[10] W. H. Kan, Y. Nadot, M. Foley, L. Ridosz, G. Proust, and J. M. Cairney, “Factors that affect the properties of additively-manufactured AlSi10Mg: Porosity versus microstructure,” *Addit Manuf*, vol. 29, 2019, doi: 10.1016/j.addma.2019.100805.

[11] S. Bai, N. Perevoshchikova, Y. Sha, and X. Wu, “The effects of selective laser melting process parameters on relative density of the AlSi10Mg parts and suitable procedures of the archimedes method,” *Applied Sciences* , vol. 9, no. 3, 2019, doi: 10.3390/app9030583.

[12] L. Wang, S. Wang, and J. Wu, “Experimental investigation on densification behavior and surface roughness of AlSi10Mg powders produced by selective laser melting,” *Opt Laser Technol*, vol. 96, pp. 88–96, Nov. 2017, doi: 10.1016/j.optlastec.2017.05.006.

[13] O. Poncelet *et al.*, “Critical assessment of the impact of process parameters on vertical roughness and hardness of thin walls of AlSi10Mg processed by laser powder bed fusion,” *Addit Manuf*, vol. 38, p. 101801, 2021, doi: 10.1016/j.addma.2020.101801.

[14] S. M. Yusuf, M. Hoegden, and N. Gao, “Effect of sample orientation on the microstructure and microhardness of additively manufactured AlSi10Mg processed by high-pressure torsion,” *The International Journal of Advanced Manufacturing Technology*, vol. 106, no. 9–10, pp. 4321–4337, 2020, doi: 10.1007/s00170-019-04817-5.

[15] A. Tridello *et al.*, “Effect of microstructure, residual stresses and building orientation on the fatigue response up to 109 cycles of an SLM AlSi10Mg alloy,” *Int J Fatigue*, vol. 137, p. 105659, 2020, doi: 10.1016/j.ijfatigue.2020.105659.

[16] B. J. Mfusi, L. C. Tshabalala, A. P. I. Popoola, and N. R. Mathe, “The effect of selective laser melting build orientation on the mechanical properties of AlSi10Mg parts,” *IOP Conf Ser Mater Sci Eng*, vol. 430, p. 012028, 2018, doi: 10.1088/1757-899X/430/1/012028.

[17] H. Abdulla, M. Maalouf, I. Barsoum, and H. An, “Truncated Newton Kernel Ridge Regression for Prediction of Porosity in Additive Manufactured SS316L,” *Applied Sciences*, vol. 12, no. 9, 2022, doi: 10.3390/app12094252.

[18] G. O. Barrionuevo, J. A. Ramos-Grez, M. Walczak, and C. A. Betancourt, “Comparative evaluation of supervised machine learning algorithms in the prediction of the relative density of 316L stainless steel fabricated by selective laser melting,” *The International Journal of Advanced Manufacturing Technology*, vol. 113, no. 1–2, pp. 419–433, 2021, doi: 10.1007/s00170-021-06596-4.

[19] C. U. Brown *et al.*, “The effects of laser powder bed fusion process parameters on material hardness and density for nickel alloy 625,” Gaithersburg, MD, 2018. doi: 10.6028/NIST.AMS.100-19.

[20] D. Wang, J. Lv, X. Wei, D. Lu, and C. Chen, “Study on Surface Roughness Improvement of Selective Laser Melted Ti6Al4V Alloy,” *Crystals (Basel)*, vol. 13, no. 2, 2023, doi: 10.3390/cryst13020306.

[21] S. Dingal, T. R. Pradhan, J. K. S. Sundar, A. R. Choudhury, and S. K. Roy, “The application of Taguchi’s method in the experimental investigation of the laser sintering process,” *The International Journal of Advanced Manufacturing Technology*, vol. 38, no. 9–10, pp. 904–914, 2008, doi: 10.1007/s00170-007-1154-1.

[22] E. Abele, H. A. Stoffregen, M. Kniepkamp, S. Lang, and M. Hampe, “Selective laser melting for manufacturing of thin-walled porous elements,” *J Mater Process Technol*, vol. 215, pp. 114–122, 2015, doi: 10.1016/j.jmatprotec.2014.07.017.

[23] J. Sun, Y. Yang, and D. Wang, “Parametric optimization of selective laser melting for forming Ti6Al4V samples by Taguchi method,” *Opt Laser Technol*, vol. 49, pp. 118–124, 2013, doi: 10.1016/j.optlastec.2012.12.002.

[24] B. Vandenbroucke and J. Kruth, “Selective laser melting of biocompatible metals for rapid manufacturing of medical parts,” *Rapid Prototyp J*, vol. 13, no. 4, pp. 196–203, 2007, doi: 10.1108/13552540710776142.
